# Supplementary material for: Validation of the accuracy of the modified World Federation of Neurosurgical Societies subarachnoid hemorrhage grading scale for predicting the outcomes of patients with aneurysmal subarachnoid hemorrhage
Source: PLoS One. 2023 Aug 22;18(8):e0289267. doi: 10.1371/journal.pone.0289267 (PMC10443875; doi:10.1371/journal.pone.0289267)
Supplement: S1 File — (PDF) [file pone.0289267.s001.pdf]

SUPPLEMENTARY RESULTS

Article title: Validation of the accuracy of the modified World Federation of Neurosurgical Societies subarachnoid hemorrhage grading scale for predicting the outcomes of patients with aneurysmal subarachnoid hemorrhage

TABLE OF CONTENT

S1 Table. Criteria per grading scale for patients with subarachnoid hemorrhage

S2 Table. Demographic and baseline characteristics of patients with aneurysmal subarachnoid hemorrhage according to neurologic function on day 30th after ictus

S3 Table. Clinical presentation, neuroimaging findings, and laboratory investigations of patients with aneurysmal subarachnoid hemorrhage according to neurologic function on day 30th after ictus

S4 Table. Initial severity of aneurysmal subarachnoid hemorrhage in patients with aneurysmal subarachnoid hemorrhage according to neurologic function on day 30th after ictus

S5 Table. Management, complications, and outcomes of patients with aneurysmal subarachnoid hemorrhage according to neurologic function on day 30th after ictus

S6 Table. Demographic and baseline characteristics of patients with aneurysmal subarachnoid hemorrhage according to neurologic function on day 90th after ictus

S7 Table. Clinical presentation, neuroimaging findings, and laboratory investigations of patients with aneurysmal subarachnoid hemorrhage according to neurologic function on day 90th after ictus

S8 Table. Initial severity of aneurysmal subarachnoid hemorrhage in patients with aneurysmal subarachnoid hemorrhage according to neurologic function on day 90th after ictus

S9 Table. Management, complications, and outcomes of patients with aneurysmal subarachnoid hemorrhage according to neurologic function on day 90th after ictus

S10 Table. Summary table of various diagnostic indices and AUROC of the modified WFNS, WFNS, and H&H scales for predicting the poor outcome (mRS of 4 to 6) after ictus in patients with aneurysmal SAH

S11 Table. Factors associated with poor outcome (mRS of 4 to 6) on day 30th after ictus in patients with aneurysmal subarachnoid hemorrhage (the exposure variable was defined as the originally-suggested 5-category modified WFNS grading scale)

S12 Table. Factors associated with poor outcome (mRS of 4 to 6) on day 30th days ictus in patients with aneurysmal subarachnoid hemorrhage (the exposure variable was defined as the originally-suggested 5-category WFNS grading scale)

S13 Table. Factors associated with poor outcome (mRS of 4 to 6) on day 30th after ictus in patients with aneurysmal subarachnoid hemorrhage (the exposure variable was defined as the originally-suggested 5-category H&H grading scale)

S14 Table. Factors associated with poor outcome (mRS of 4 to 6) on day 90th after ictus in patients with aneurysmal subarachnoid hemorrhage (the exposure variable was defined as the originally-suggested 5-category modified WFNS grading scale)

S15 Table. Factors associated with poor outcome (mRS of 4 to 6) on day 90th after ictus in patients with aneurysmal subarachnoid hemorrhage (the exposure variable was defined as the originally-suggested 5-category WFNS grading scale)

S 16 Table. Factors associated with poor outcome (mRS of 4 to 6) on day 90th after ictus in patients with aneurysmal subarachnoid haemorrhage (the exposure variable was defined as the originally-suggested 5-category H&H grading scale)

S17 Table. Factors associated with poor outcome (mRS of 4 to 6) on day 30th after ictus in patients with aneurysmal subarachnoid haemorrhage (the exposure variable was defined as the grades of the WFNS scale which were higher than or equal to the cut-off value)

S18 Table. Factors associated with poor outcome (mRS of 4 to 6) on day 30th after ictus in patients with aneurysmal subarachnoid haemorrhage (the exposure variable was defined as the grades of the H&H scale which were higher than or equal to the cut-off value)

S19 Table. Factors associated with poor outcome (mRS of 4 to 6) on day 90th after ictus in patients with aneurysmal subarachnoid haemorrhage (the exposure variable was defined as the grades of the WFNS scale which were higher than or equal to the cut-off value)

S20 Table. Factors associated with poor outcome (mRS of 4 to 6) on day 90th after ictus in patients with aneurysmal subarachnoid haemorrhage (the exposure variable was defined as the grades of the H&H scale which were higher than or equal to the cut-off value)

S21 Table. Breakdown of missing data

SUPPLEMENTARY TABLES

S1 Table. Criteria per grading scale for patients with subarachnoid hemorrhage

Numerical SAH grading scales (point)	Descriptive SAH grading scales			
	Grade	H&H grading scale (Hunt WE. *J Neurosurg*. 1968)	WFNS grading scale (WFNS. *J Neurosurg*. 1988)	Modified WFNS scale (Sano H. *World Neurosurg*. 2015)
1	I	Asymptomatic or mild headache and slight nuchal rigidity	GCS score of 15, no motor deficit	GCS score of 15
2	II	Severe headache, stiff neck, no neurologic deficit except cranial nerve palsy	GCS score of 13 to 14, no motor deficit	GCS score of 14
3	III	Drowsy or confused, mild focal neurologic deficit	GCS score of 13 to 14, motor deficit	GCS score of 13
4	IV	Stuporous, moderate or severe hemiparesis	GCS score of 7 to 12	GCS score of 7 to 12
5	V	Coma, decerebrate posturing	GCS score of 3 to 6	GCS score of 3 to 6

Abbreviations

GCS: Glasgow coma scale; **H&H**: Hunt and Hess; **SAH**, subarachnoid hemorrhage; **WFNS**: World Federation of Neurological Surgeons

S2 Table. Demographic and baseline characteristics of patients with aneurysmal subarachnoid haemorrhage according to neurologic function on day 30th after ictus

	All cases	mRS of 0 to 3	mRS of 4 to 6	p-value[a]
Prehospital setting	n=415	n=277	n=138	
Transferred from local hospitals, n (%)	345 (83.1)	234 (84.5)	111 (80.4)	0.300
Hospital taken to, n (%)	n=415	n=277	n=138	0.105
Viet Duc	30 (7.2)	25 (9.0)	5 (3.6)	
Bach Mai	358 (86.3)	236 (85.2)	122 (88.4)	
Hanoi Medical University	27 (6.5)	16 (5.8)	11 (8.0)	
**Demographics**	n=415	n=277	n=138	
Age (year), median (IQR)	57.0 (48.0-67.0)	56.0 (46.5-64.0)	63.5 (52.0-72.0)	<0.001[***]
Gender (male), no. (%)	198 (47.7)	131 (47.3)	67 (48.6)	0.809
**Risk factors for aneurysmal SAH**	n=415	n=277	n=138	
Cigarette smoking, no. (%)	159 (38.3)	102 (36.8)	57 (41.3)	0.376
Hypertension, no. (%), n=413	163 (39.5)	93 (33.7)	70 (51.1)	0.001
Genetic risk, no. (%)	6 (1.4)	5 (1.8)	1 (0.7)	0.668[*]
Alcohol consumption, no. (%), n=401	182 (45.4)	117 (44.2)	65 (47.8)	0.448
Sympathomimetic drugs, no. (%), n=413	3 (0.7)	3 (1.1)	0 (0.0)	0.554[*]
Estrogen deficiency[b], no. (%), n=213	33 (15.5)	20 (14.1)	13 (18.3)	0.422
Antithrombotic therapy, no. (%)	4 (1.0)	1 (0.4)	3 (2.2)	0.109[*]
Elevated total cholesterol, no. (%)	11 (2.7)	6 (2.2)	5 (3.6)	0.517[*]
**Comorbidities**	n=415	n=277	n=138	
Cerebrovascular disease, no. (%)	18 (4.3)	10 (3.6)	8 (5.8)	0.303
Chronic cardiac failure, no. (%)	7 (1.7)	3 (1.1)	4 (2.9)	0.228[*]
Coronary artery disease/MI, no. (%)	7 (1.7)	3 (1.1)	4 (2.9)	0.228[*]
COPD/Asthma, no. (%)	5 (1.2)	3 (1.1)	2 (1.4)	>0.999[*]
Active neoplasm, no. (%)	7 (1.7)	5 (1.8)	2 (1.4)	>0.999[*]
Chronic renal failure, no.	3 (0.7)	2 (0.7)	1 (0.7)	>0.999[*]

(%)				
Ulcer disease, no. (%)	4 (1.0)	2 (0.7)	2 (1.4)	0.603[*]
Diabetes mellitus, no. (%)	27 (6.5)	10 (3.6)	17 (12.3)	0.001
Hematological disease, no. (%)	2 (0.5)	1 (0.4)	1 (0.7)	>0.999[*]

[a] Comparison between mRS of 0 to 3 and mRS of 4 to 6 using Chi-squared test; *Fisher's exact test; **Mann–Whitney U test; *** Independent Samples T test

[b]Exclude 198 male patients and 4 female patients with missing data

Abbreviations: **COPD**: chronic obstructive pulmonary disease; **IQR**: interquartile range; **MI**: myocardial ischemia; **mRS**: modified Rankin Scale; no.: number; **SAH**: subarachnoid hemorrhage.

S3 Table. Clinical presentation, neuroimaging findings, and laboratory investigations of patients with aneurysmal subarachnoid haemorrhage according to neurologic function on day 30th after ictus

	All cases	mRS of 0 to 3	mRS of 4 to 6	p-value[a]
**Onset symptoms**	n=415	n=277	n=138	
Sudden-onset, severe headache, no. (%)	337 (81.2)	252 (91.0)	85 (61.6)	<0.001
Vomiting, no. (%)	248 (59.8)	189 (68.2)	59 (42.8)	<0.001
Neck pain or stiffness, no. (%)	111 (26.7)	83 (30.0)	28 (20.3)	0.036
Photophobia, no. (%)	15 (3.6)	13 (4.7)	2 (1.4)	0.16[*]
Blurred or double vision, no. (%)	12 (2.9)	10 (3.6)	2 (1.4)	0.352[*]
Brief loss of consciousness, no. (%)	146 (35.2)	62 (22.4)	84 (60.9)	<0.001
Seizures, no. (%)	18 (4.3)	10 (3.6)	8 (5.8)	<0.303
**Clinical presentation on admission**	n=415	n=277	n=138	
GCS score, median (IQR)	14.0 (10.0-15.0)	15.0 (14.0-15.0)	8.0 (6.0-12.25)	<0.001[**]
Focal deficits, no. (%)	315(75.9)	197 (71.1)	118 (85.5)	0.001
Focal signs, n (%)				
Third nerve palsy	8 (2.5)	4 (2.0)	4 (3.4)	0.479[*]
Sixth nerve palsy	1 (0.3)	1 (0.5)	0 (0.0)	>0.999[*]
Hemiparesis	59 (18.7)	33 (16.8)	26 (22.0)	0.245
Aphasia	10 (3.2)	5 (2.5)	5 (4.2)	0.510[*]
Bilateral leg weakness	2 (0.6)	1 (0.5)	1 (0.8)	>0.999[*]
Ophthalmoplegia	1 (0.3)	1 (0.5)	0 (0.0)	>0.999[*]
Impaired level of consciousness, n=314	154 (49.0)	58 (29.4)	96 (82.1)	<0.001
Brainstem signs	5 (1.6)	1 (0.5)	4 (3.4)	0.067[*]
Neck stiffness	254 (80.6)	168 (85.3)	86 (72.9)	0.007
**Neuroimaging findings on admission**	n=415	n=277	n=138	

Blood filling the subarachnoid space, no. (%)				
Basal cistern, n=411	228 (55.5)	132 (47.8)	96 (71.1)	<0.001
Sylvian fissure, n=413	380 (92.0)	248 (89.9)	132 (96.4)	0.22
Interhemispheric fissure, n=412	291 (70.6)	183 (66.3)	108 (79.4)	0.006
Interpeduncular fossa, n=412	266 (64.6)	156 (56.5)	110 (80.9)	<0.001
Suprasellar cistern, n=412	270 (65.5)	168 (60.9)	102 (75.0)	0.005
Ambient cistern, n=412	258 (62.6)	151 (54.7)	107 (78.7)	<0.001
Quadrigeminal cistern, n=412	126 (30.6)	52 (18.8)	74 (54.4)	<0.001
IVH, n (%)	275 (66.3)	163 (58.8)	112 (81.2)	<0.001
ICH, n (%)	85 (20.5)	47 (17.0)	38 (27.5)	0.012
ICH volume (mL), mean (SD), n=85	22.6 (22.82)	17.55 (16.98)	28.86 (27.40)	0.097**
Subdural hemorrhage, n (%)	21 (5.1)	9 (3.2)	12 (8.7)	0.017
Hydrocephalus, n (%)	133 (32.0)	66 (23.8)	67 (48.6)	<0.001
Evans' index, mean (SD), n=392	0.27 (0.07)	0.27 (0.07)	0.29 (0.08)	0.002**
Hypodense lesions on computed tomography, n (%), n=413	26 (6.3)	9 (3.3)	17 (12.4)	<0.001
Aneurysm site, n (%)				
Internal carotid artery (ICA)	84 (20.2)	55 (19.9)	29 (21.0)	0.782
Ophtalmic segment of the ICA (OphIC)	3 (0.7)	3 (1.1)	0 (0.0)	0.554*
Cavernous segment of the ICA (cIC)	7 (1.7)	5 (1.8)	2 (1.4)	>0.999*
Anterior choroidal artery segment of the ICA (AchIC)	1 (0.2)	1 (0.4)	0 (0.0)	>0.999*
Posterior communicating artery (PCoA)	65 (15.7)	50 (18.1)	15 (10.9)	0.058
Anterior cerebral artery (ACA)	31 (7.5)	21 (7.6)	10 (7.2)	0.903
Anterior communicating artery (AcoA)	130 (31.3)	85 (30.7)	45 (32.6)	0.691

Middle cerebral artery (MCA)	85 (20.5)	62 (22.4)	23 (16.7)	0.174
Posterior cerebral artery (PCA)	1 (0.2)	1 (0.4)	0 (0.0)	>0.999*
Vertebral artery (VA)	18 (4.3)	7 (2.5)	11 (8.0)	0.010
Superior cerebellar artery (SCA)	0 (0.0)	0 (0.0)	0 (0.0)	NA
Posterior inferior cerebellar artery (PICA)	9 (2.2)	5 (1.8)	4 (2.9)	0.488*
Anterior inferior cerebellar artery (AICA)	0 (0.0)	0 (0.0)	0 (0.0)	NA
Basilar artery (BA)	15 (3.6)	9 (3.2)	6 (4.3)	0.584*
**Admission laboratory investigations**				
Platelets (G/L), mean (SD), n=408	261.47 (76.04)	257.86 (72.65)	268.7 (82.20)	0.217**
PT-INR, mean (SD), n=398	1.03 (0.51)	1.03 (0.62)	1.04 (0.17)	0.036**

[a] Comparison between mRS of 0 to 3 and mRS of 4 to 6 using Chi-squared test; *Fisher's exact test; **Mann–Whitney U test.

Abbreviations: **GCS**: Glasgow coma scale; **ICH**: intracerebral hemorrhage; **IQR**: interquartile range; **IVH**: intraventricular hemorrhage; **mRS**: modified Rankin Scale; **no.**: number; **PT-INR**: prothrombin time with international normalized ratio; **SD**: standard deviation.

S4 Table. Initial severity of aneurysmal subarachnoid haemorrhage in patients with aneurysmal subarachnoid haemorrhage according to neurologic function on day 30th after ictus

	All cases (n=415)	mRS of 0 to 3 (n=277)	mRS of 4 to 6 (n=138)	p-value[a]
Modified WFNS score, median (IQR)	2.0 (1.0-4.0)	1.0 (1.0-2.0)	4.0 (3.75-5.0)	<0.001**
Modified WFNS scale, no. (%)				<0.001
Grade I	204 (49.2)	185 (66.8)	19 (13.8)	
Grade II	38 (9.2)	33 (11.9)	5 (3.6)	
Grade III	24 (5.8)	14 (5.1)	10 (7.2)	
Grade IV	99 (23.9)	38 (13.7)	61 (44.2)	
Grade V	50 (12.0)	7 (2.5)	43 (31.2)	
WFNS score, median (IQR)	2.0 (1.0-4.0)	1.0 (1.0-2.0)	4.0 (3.75-5.0)	<0.001**
WFNS scale, no. (%)				<0.001
Grade I	204 (49.2)	185 (66.8)	19 (13.8)	
Grade II	48 (11.6)	37 (13.4)	11 (8.0)	
Grade III	14 (3.4)	10 (3.6)	4 (2.9)	

Grade IV	99 (23.9)	38 (13.7)	61 (44.2)	
Grade V	50 (12.0)	7 (2.5)	43 (31.2)	
H&H score, median (IQR)	2.0 (2.0-4.0)	2.0 (2.0-3.0)	5.0 (3.0-5.0)	<0.001**
H&H scale, no. (%)				<0.001
Grade I	45 (10.8)	40 (14.4)	5 (3.6)	
Grade II	168 (40.5)	153 (55.2)	15 (10.9)	
Grade III	62 (14.9)	43 (15.5)	19 (13.8)	
Grade IV	48 (11.6)	25 (9.0)	23 (16.7)	
Grade V	92 (22.2)	16 (5.8)	76 (55.1)	
Fisher score, median (IQR), n=414	4.0 (3.0-4.0)	4.0 (3.0-4.0)	4.0 (4.0-4.0)	<0.001**
Fisher scale, no. (%)	n=414	n=276	n=138	<0.001*
Group 1	2 (0.5)	1 (0.4)	1 (0.7)	
Group 2	24 (5.8)	23 (8.3)	1 (0.7)	
Group 3	98 (23.7)	80 (29.0)	18 (13.0)	
Group 4	290 (70.0)	172 (62.3)	118 (85.5)	

[a] Comparison between mRS of 0 to 3 and mRS of 4 to 6 using Chi-squared test; *Fisher's exact test; **Mann–Whitney U test.

[b] The descriptive SAH grading scales (i.e., the modified WFNS, WFNS, and H&H scales) were converted to the numerical SAH grading scales in ascending order (see S1 Table in S1 File for additional information).

Abbreviations: **H&H**: Hunt and Hess; **IQR**: interquartile range; **mRS**: modified Rankin Scale; **no.**: number; **SD**: standard deviation; **WFNS**: World Federation of Neurological Surgeons.

S5 Table. Management, complications, and outcomes of patients with aneurysmal subarachnoid hemorrhage according to neurologic function on day 30th after ictus

	All cases (n=415)	mRS of 0 to 3 (n=277)	mRS of 4 to 6 (n=138)	p-value[a]
**Aneurysm repairs and other treatments**	n=415	n=277	n=138	
No aneurysm repair, no. (%)	74 (17.8)	4 (1.4)	70 (50.7)	<0.001
Endovascular coiling, no. (%)	169 (40.7)	146 (52.7)	23 (16.7)	<0.001
Surgical clipping, no. (%)	172 (41.5)	127 (45.8)	45 (32.6)	0.010
Surgical hematoma evacuation[b], no. (%)	44 (10.6)	21 (7.6)	23 (16.7)	0.005
EVD[c], no. (%), n=414	43 (10.4)	17 (6.2)	26 (18.8)	<0.001
IVF, no. (%)	3 (0.7)	1 (0.4)	2 (1.4)	0.258*
Nimodipine, no. (%), n=363	331 (91.2)	235 (97.9)	96 (78.0)	<0.001
**Complications**				
Rebleeding, no. (%), n=411	18 (4.4)	4 (1.4)	14 (10.4)	<0.001
Early rebleeding, no. (%), n=13	1 (7.7)	1 (33.3)	0 (0.0)	0.231*
Late rebledding, no.	12 (92.3)	2 (66.7)	10 (100)	0.231*

(%), n=13				
DCI, no. (%), n=409	25 (6.1)	6 (2.2)	19 (14.1)	<0.001
Acute hydrocephalus, no. (%)	136 (32.8)	67 (24.2)	69 (50.0)	<0.001
Hyponatremia, no. (%)	71 (17.1)	45 (16.2)	26 (18.8)	0.508
Seizures, no. (%)	53 (12.8)	36 (13.0)	17 (12.3)	0.846
Chronic hydrocephalus, no. (%), n=311	8 (2.6)	6 (2.6)	2 (2.4)	>0.999*
Ventriculitis, no. (%), n=369	13 (3.5)	5 (2.0)	8 (6.5)	0.037*
Pneumonia, no. (%)	58 (14.0)	22 (7.9)	36 (26.1)	<0.001
Urinary tract infection, no. (%)	9 (2.2)	4 (1.4)	5 (3.6)	0.166*
**Clinical time course**	n=415	n=277	n=138	
Ictus to hospital arrival (hour), no. (%), n=408				0.060
≤ 24 hours	212 (52.0)	131 (48.2)	81 (59.6)	
>24–72 hours	188 (46.0)	134 (49.2)	54 (39.7)	
>72 hours	8 (2.0)	7 (2.6)	1 (0.7)	
Length of hospitalization (days), mean (SD)	10.14 (9.85)	11.11 (9.41)	8.2 (10.46)	<0.001**
**Clinical outcomes**	n=415	n=277	n=138	
Hospital discharge, no. (%)	119 (28.7)	115 (41.5)	4 (2.9)	<0.001
Transferred to another hospital, no. (%)	252 (60.7)	162 (58.5)	90 (65.2)	0.186
Discharged to die, no. (%)	33 (8.0)	1 (0.4)	32 (23.2)	<0.001
Deaths:				
Died in hospital, no. (%)	71 (17.1)	0 (0.0)	71 (51.4)	<0.001
Died within 30 days of ictus, no. (%)	89 (21.4)	0 (0.0)	89 (64.5)	<0.001
Neurological function:				
mRS score at hospital discharge, median (IQR)	1.0 (1.0-5.0)	1.0 (1.0-1.0)	5.0 (5.0-5.0)	<0.001**
mRS at hospital discharge, no. (%)				<0.001
Good (mRS of 0 to 3)	266 (64.1)	262 (94.6)	4 (2.9)	
Poor (mRS of 4 to 6)	149 (35.9)	15 (5.4)	134 (97.1)	

[a] Comparison between mRS of 0 to 3 and mRS of 4 to 6 using Chi-squared test; *Fisher's exact test; **Mann–Whitney U test.

[b] Surgical hematoma evacuation was defined as any surgical procedure evacuating epidural, subdural, intraventricular, or intraparenchymal haematoma, such as decompressive craniotomy, open craniotomy, or minimally invasive surgery.

[c] Data on the reason for the insertion of an EVD was acute hydrocephalus which accounted for only 7.7%

(32/414) of patients with aneurysmal SAH.
Abbreviations: **DCI**: delayed cerebral ischemia; **EVD**: external ventricular drainage; **IQR**: interquartile range; **IVF**: intraventricular fibrinolysis; **mRS**: modified Rankin Scale; **no.**: number; **SD**: standard deviation.

S6 Table. Demographic and baseline characteristics of patients with aneurysmal subarachnoid haemorrhage according to neurologic function on day 90th after ictus

	All cases	mRS of 0 to 3	mRS of 4 to 6	p-value[a]
Prehospital setting	n=415	n=282	n=133	
Transferred from local hospitals, no. (%)	345 (83.1)	238 (84.4)	107 (80.5)	0.316
Hospital taken to, n (%)	n=415	n=282	n=133	0.052
Viet Duc	30 (7.2)	26 (9.2)	4 (3.0)	
Bach Mai	358 (86.3)	240 (85.1)	118 (87.1)	
Hanoi Medical University	27 (6.5)	16 (5.7)	11 (8.3)	
**Demographics**	n=415	n=282	n=133	
Age (year), median (IQR)	57.0 (48.0-67.0)	56.0 (46.0-64.0)	64.0 (53.0-72.5)	<0.001***
Gender (male), no. (%)	198 (47.7)	134 (47.5)	64 (48.1)	0.909
**Risk factors for aneurysmal SAH**	n=415	n=282	n=133	
Cigarette smoking, no. (%)	159 (38.3)	103 (36.5)	56 (42.1)	0.275
Hypertension, no. (%), n=413	163 (39.5)	94 (33.6)	69 (51.9)	<0.001
Genetic risk, no. (%)	6 (1.4)	5 (1.8)	1 (0.8)	0.669*
Alcohol consumption, no. (%), n=401	182 (45.4)	119 (44.1)	63 (48.1)	0.449
Sympathomimetic drugs, no. (%), n=413	3 (0.7)	3 (1.1)	0 (0.0)	0.554*
Estrogen deficiency[b], no. (%), n=213	33 (15.5)	20 (14.1)	13 (18.3)	0.422
Antithrombotic therapy, no. (%)	4 (1.0)	1 (0.4)	3 (2.3)	0.099*
Elevated total cholesterol, no. (%)	11 (2.7)	6 (2.1)	5 (3.8)	0.340*
**Comorbidities**	n=415	n=282	n=133	
Cerebrovascular disease, no. (%)	18 (4.3)	10 (3.5)	8 (6.0)	0.249
Chronic cardiac failure, no. (%)	7 (1.7)	3 (1.1)	4 (3.0)	0.218*
Coronary artery disease/MI, no. (%)	7 (1.7)	3 (1.1)	4 (3.0)	0.218*

COPD/Asthma, no. (%)	5 (1.2)	3 (1.1)	2 (1.5)	0.657*
Active neoplasm, no. (%)	7 (1.7)	5 (1.8)	2 (1.5)	>0.999*
Chronic renal failure, no. (%)	3 (0.7)	2 (0.7)	1 (0.8)	>0.999*
Ulcer disease, no. (%)	4 (1.0)	2 (0.7)	2 (1.5)	0.596
Diabetes mellitus, no. (%)	27 (6.5)	10 (3.5)	17 (12.8)	<0.001
Hematological disease, no. (%)	2 (0.5)	1 (0.4)	1(0.8)	0.539*

[a]Comparison between mRS of 0 to 3 and mRS of 4 to 6 using Chi-squared test; *Fisher's exact test; **Mann–Whitney U test; *** Independent Samples T test.

[b]Exclude 198 male patients and 4 female patients with missing data.

Abbreviations: **COPD**: chronic obstructive pulmonary disease; **IQR**: interquartile range; **MI**: myocardial ischemia; **mRS**: modified Rankin Scale; no.: number; **SAH**: subarachnoid hemorrhage.

S7 Table. Clinical presentation, neuroimaging findings, and laboratory investigations of patients with aneurysmal subarachnoid haemorrhage according to neurologic function on day 90th after ictus

	All cases	mRS of 0 to 3	mRS of 4 to 6	p-value[a]
**Onset symptoms**	n=415	n=282	n=133	
Sudden-onset, severe headache, no. (%)	337 (81.2)	255 (90.4)	82 (61.7)	<0.001
Vomiting, no. (%)	248 (59.8)	190 (67.4)	58 (43.6)	<0.001
Neck pain or stiffness, no. (%)	111 (26.7)	84 (29.8)	27 (20.3)	0.042
Photophobia, no. (%)	15 (3.6)	13 (4.6)	2 (1.5)	0.160
Blurred or double vision, no. (%)	12 (2.9)	10 (3.5)	2 (1.5)	0.353
Brief loss of consciousness, no. (%)	146 (35.2)	65 (23.0)	81 (60.9)	<0.001
Seizures, no. (%)	18 (4.3)	10 (3.5)	8 (6.0)	0.249
**Clinical presentation on admission**	n=415	n=282	n=133	
GCS score, median (IQR)	14 (10.0-15.0)	15 (14.0-15.0)	8 (6.0-12.0)	<0.001**
Focal deficits, no. (%)	315 (75.9)	202 (71.6)	113 (85.0)	0.003
Focal signs, n (%)	n=315	n=202	n=113	
Third nerve palsy	8 (2.5)	5 (2.5)	3 (2.7)	>0.999*
Sixth nerve palsy	1 (0.3)	1 (0.5)	0 (0.0)	>0.999*
Hemiparesis	59 (18.7)	33 (16.3)	26 (23.0)	0.145
Aphasia	10 (3.2)	5 (2.5)	5 (4.4)	0.339*
Bilateral leg weakness	2 (0.6)	1 (0.5)	1 (0.9)	>0.999*
Ophthalmoplegia	1 (0.3)	1 (0.5)	0 (0.0)	>0.999*
Impaired level of consciousness, n=314	154 (49.0)	62 (30.7)	92 (82.1)	<0.001

Brainstem signs	5 (1.6)	1 (0.5)	4 (3.5)	0.058*
Neck stiffness	254 (80.6)	171 (84.7)	83 (73.5)	0.016
**Neuroimaging findings on admission**	n=415	n=282	n=133	
Location of blood within the subarachnoid space, no. (%)				
Basal cistern, n=411	228 (55.5)	135 (48.0)	93 (71.5)	<0.001
Sylvian fissure, n=413	380 (92.0)	253 (90.0)	127 (96.2)	0.031
Interhemispheric fissure, n=412	291 (70.6)	186 (66.2)	105 (80.2)	0.004
Interpeduncular fossa, n=412	266 (64.6)	160 (56.9)	106 (80.9)	<0.001
Suprasellar cistern, n=412	270 (65.5)	172 (61.2)	98 (74.8)	0.007
Ambient cistern, n=412	258 (62.6)	155 (55.2)	103 (78.6)	<0.001
Quadrigeminal cistern, n=412	126 (30.6)	53 (18.9)	73 (55.7)	<0.001
IVH, no. (%)	275 (66.3)	168 (59.6)	107 (80.5)	<0.001
ICH, no. (%)	85 (20.5)	48 (17.0)	37 (27.8)	0.011
ICH volume (mL), mean (SD), n=85	22.6 (22.82)	17.79 (16.88)	28.85 (27.78)	0.134**
Subdural hemorrhage, no. (%)	21(5.1)	9 (3.2)	12 (9.0)	0.011
Hydrocephalus, no. (%)	133(32.0)	67 (23.8)	66 (49.6)	<0.001
Evans' index, mean (SD), n=392	0.27 (0.07)	0.26 (0.07)	0.29 (0.08)	0.001**
Hypodense lesions on computed tomography, no. (%), n=413	26 (6.3)	9 (3.2)	17 (12.9)	<0.001
Aneurysm site, n (%)				
Internal carotid artery (ICA)	84 (20.2)	56 (19.9)	28 (21.1)	0.777
Ophtalmic segment of the ICA (OphIC)	3 (0.7)	3 (1.1)	0 (0.0)	0.554*
Cavernous segment of the ICA (cIC)	7 (1.7)	5 (1.8)	2 (1.5)	>0.999*
Anterior choroidal artery segment of the ICA (AchIC)	1 (0.2)	1 (0.4)	0 (0.0)	>0.999*
Posterior communicating artery (PCoA)	65 (15.7)	50 (17.7)	15 (11.3)	0.091
Anterior cerebral	31 (7.5)	21 (7.4)	10 (7.5)	0.979

artery (ACA)				
Anterior communicating artery (AcoA)	130 (31.3)	86 (30.5)	44 (33.1)	0.596
Middle cerebral artery (MCA)	85 (20.5)	64 (22.7)	21 (15.8)	0.104
Posterior cerebral artery (PCA)	1 (0.2)	1 (0.4)	0 (0.0)	>0.999*
Vertebral artery (VA)	18 (4.3)	7 (2.5)	11 (8.3)	0.007
Superior cerebellar artery (SCA)	0 (0.0)	0 (0.0)	0 (0.0)	NA
Posterior inferior cerebellar artery (PICA)	9 (2.2)	5 (1.8)	4 (3.0)	0.476*
Anterior inferior cerebellar artery (AICA)	0 (0.0)	0 (0.0)	0 (0.0)	NA
Basilar artery (BA)	15 (3.6)	10 (3.5)	5 (3.8)	>0.999*
**Admission laboratory investigations**				
Platelets (G/L), mean (SD), n=408	261.47 (76.04)	257.41 (72.27)	270.07 (83.10	0.132**
PT-INR, mean (SD), n=398	1.03 (0.51)	1.03 (0.61)	1.03 (0.17)	0.042**

[a]Comparison between mRS of 0 to 3 and mRS of 4 to 6 using Chi-squared test; *Fisher's exact test; **Mann–Whitney U test.

Abbreviations: **GCS**: Glasgow coma scale; **ICH**: intracerebral hemorrhage; **IQR**: interquartile range; **IVH**: intraventricular hemorrhage; **mRS**: modified Rankin Scale; **no.**: number; **PT-INR**: prothrombin time with international normalized ratio; **SD**: standard deviation.

S8 Table. Initial severity of aneurysmal subarachnoid haemorrhage in patients with aneurysmal subarachnoid haemorrhage according to neurologic function on day 90th after ictus

	All cases (n=415)	mRS of 0 to 3 (n=282)	mRS of 4 to 6 (n=133)	p-value[a]
Modified WFNS score[b], median (IQR)	2.0 (1.0-4.0)	1.0 (1.0-2.0)	4.0 (4.0-5.0)	<0.001**
Modified WFNS scale, no. (%)				<0.001
Grade I	204 (49.2)	186 (66.0)	18 (13.5)	
Grade II	38 (9.2)	33 (11.7)	5 (3.8)	
Grade III	24 (5.8)	15 (5.3)	9 (6.8)	
Grade IV	99 (23.9)	41 (14.5)	58 (43.6)	
Grade V	50 (12.0)	7 (2.5)	43 (32.3)	
WFNS score[b], median	2.0 (1.0-4.0)	1.0 (1.0-2.0)	4.0 (4.0-5.0)	<0.001**

(IQR)				
WFNS scale, no. (%)				<0.001
Grade I	204 (49.2)	186 (66.0)	18 (13.5)	
Grade II	48 (11.6)	38 (13.5)	10 (7.5)	
Grade III	14 (3.4)	10 (3.5)	4 (3.0)	
Grade IV	99 (23.9)	41 (14.5)	58 (43.6)	
Grade V	50 (12.0)	7 (2.5)	43 (32.3)	
H&H score[b], median (IQR)	2.0 (2.0-4.0)	2.0 (2.0-3.0)	5.0 (3.0-5.0)	<0.001[**]
H&H scale, no. (%)				<0.001
Grade I	45 (10.8)	40 (12.2)	5 (3.8)	
Grade II	168 (40.5)	154 (54.6)	14 (10.5)	
Grade III	62 (14.9)	44 (15.6)	18 (13.5)	
Grade IV	48 (11.6)	26 (9.2)	22 (16.5)	
Grade V	92 (22.2)	18 (6.4)	74 (55.6)	
Fisher score[b], median (IQR), n=414	4.0 (3.0-4.0)	4.0 (3.0-4.0)	4.0 (4.0-4.0)	<0.001[**]
Fisher scale, no. (%)	n=414	n=281	n=133	<0.001
Group 1	2 (0.5)	1 (0.4)	1 (0.8)	
Group 2	24 (5.8)	23 (8.2)	1 (0.8)	
Group 3	98 (23.7)	80 (28.5)	18 (13.5)	
Group 4	290 (70.0)	177 (63.0)	113 (85.0)	

[a] Comparison between mRS of 0 to 3 and mRS of 4 to 6 using Chi-squared test; *Fisher's exact test; **Mann–Whitney U test.

[b] The descriptive SAH grading scales (i.e., the modified WFNS, WFNS, and H&H scales) were converted to the numerical SAH grading scales in ascending order (see S1 Table in S1 File for additional information).

Abbreviations: **H&H**: Hunt and Hess; **IQR**: interquartile range; **mRS**: modified Rankin Scale; **no.**: number; **SD**: standard deviation; **WFNS**: World Federation of Neurological Surgeons.

S9 Table. Management, complications, and outcomes of patients with aneurysmal subarachnoid haemorrhage according to neurologic function on day 90th after ictus

	All cases	mRS of 0 to 3	mRS of 4 to 6	p-value[a]
**Aneurysm repairs and other treatments**	n=415	n=282	n=133	
No aneurysm repair, no. (%)	74 (17.8)	5 (1.8)	69 (51.9)	<0.001
Endovascular coiling, no. (%)	169 (40.7)	147 (52.1)	22 (16.5)	<0.001
Surgical clipping, no. (%)	172 (41.5)	130 (46.1)	42 (31.6)	0.005
Surgical hematoma evacuation[b], no. (%)	44 (10.6)	23 (8.2)	21 (15.8)	0.018
EVD[c], no. (%), n=414	43 (10.4)	18 (6.4)	25 (18.8)	<0.001
IVF, no. (%)	3 (0.7)	1 (0.4)	2 (1.5)	0.242[*]

Nimodipine, no. (%), n=363	331 (91.2)	239 (98.0)	92 (77.3)	<0.001
**Complications**	n=415	n=282	n=133	
Rebleeding, no. (%), n=411	18 (4.4)	5 (1.8)	13 (10.0)	<0.001
Early rebleeding, no. (%), n=13	1 (7.7)	0 (0.0)	1 (8.3)	>0.999[*]
Late rebledding, no. (%), n=13	12 (92.3)	1 (100)	11 (91.7)	>0.999[*]
DCI, no. (%), n=409	25 (6.1)	7 (2.5)	18 (13.8)	<0.001
Acute hydrocephalus, no. (%)	136 (32.8)	69 (24.5)	67 (50.4)	<0.001
Hyponatremia, no. (%)	71 (17.1)	46 (16.3)	25 (18.8)	0.577
Seizures, no. (%)	53 (12.8)	37 (13.1)	16 (12.0)	0.756
Chronic hydrocephalus, no. (%), n=311	8 (2.6)	6 (2.6)	2 (2.5)	>0.999[*]
Ventriculitis, no. (%), n=369	13 (3.5)	5 (2.0)	8 (6.7)	0.032[*]
Pneumonia, no. (%)	58 (14.0)	23 (8.2)	35 (26.3)	<0.001
Urinary tract infection, no. (%)	9 (2.2)	5 (1.8)	4 (3.0)	0.476[*]
**Clinical time course**	n=415	n=282	n=133	
Ictus to hospital arrival (hour), no. (%), n=408				0.089
≤24 hours	212 (52.0)	134 (48.6)	78 (59.1)	
>24 - 72 hours	188 (46.0)	135 (48.9)	53 (40.1)	
>72 hours	8 (2.0)	7 (2.5)	1 (0.8)	
Length of hospitalization (days), mean (SD)	10.14 (9.85)	11.22 (9.5)	7.84 (10.22)	<0.001[**]
**Clinical outcomes**	n=415	n=282	n=133	
Hospital discharge, no. (%)	119 (28.7)	116 (41.1)	3 (2.3)	<0.001
Transferred to another hospital, no. (%)	252 (60.7)	166 (58.9)	86 (64.7)	0.259
Discharged to die, no. (%)	33 (8.0)	1 (0.4)	32 (24.1)	<0.001
Deaths:				
Died in hospital, no. (%)	71 (17.1)	0 (0.0)	71 (53.4)	<0.001
Died within 30 days of ictus, no. (%)	89 (21.4)	0 (0.0)	89 (66.9)	<0.001
Died within 90 days of ictus, no. (%)	97 (23.4)	0 (0.0)	97 (72.9)	<0.001
Neurological function:				
mRS score at hospital	1.0 (1.0-5.0)	1.0 (1.0-1.0)	5.0 (5.0-5.0)	<0.001[**]

discharge, median (IQR)				
mRS at hospital discharge, no. (%)				<0.001
Good (mRS of 0 to 3)	266 (64.1)	262 (92.9)	4 (3.0)	
Poor (mRS of 4 to 6)	149 (35.9)	20 (7.1)	129 (97.0)	
mRS score at 30 days of ictus, median (IQR)	2.15 (2.54)	0.51 (0.92)	5.63 (0.68)	<0.001**
mRS at 30 days of ictus, no. (%)				<0.001
Good (mRS of 0 to 3)	277 (66.7)	277 (98.2)	0 (0.0)	
Poor (mRS of 4 to 6)	138 (33.3)	5 (1.8)	133 (100)	

[a] Comparison between mRS of 0 to 3 and mRS of 4 to 6 using Chi-squared test; *Fisher's exact test; **Mann–Whitney U test.

[b] Surgical haematoma evacuation was defined as any surgical procedure evacuating epidural, subdural, intraventricular, or intraparenchymal haematoma, such as decompressive craniotomy, open craniotomy, or minimally invasive surgery.

[c] Data on the reason for the insertion of an EVD was acute hydrocephalus which accounted for only 7.7% (32/414) of patients with aneurysmal SAH.

Abbreviations: **DCI**: delayed cerebral ischemia; **EVD**: external ventricular drainage; **IQR**: interquartile range; **IVF**: intraventricular fibrinolysis; **mRS**: modified Rankin Scale; **no.**: number; **SD**: standard deviation.

S10 Table. Summary table of various diagnostic indices and AUROC of the modified WFNS, WFNS, and H&H scales for predicting the poor outcome (mRS of 4 to 6) after ictus in patients with aneurysmal SAH

Variable	Cutoff value	Youden's index J	Sensitivity (%)	Specificity (%)	LR+	LR–	AUROC (95% CI)	p-value
**Poor outcome on day 30th after ictus**								
Modified WFNS	≥2.5	0.613	82.6	78.7	3.878	0.221	0.839 (0.796-0.883)	<0.001
WFNS	≥3.5	1.591	75.4	83.8	4.639	0.294	0.836 (0.793-0.880)	<0.001
H&H	≥3.5	0.569	71.7	85.2	4.847	0.332	0.839 (0.795-0.883)	<0.001
**Poor outcome on day 90th after ictus**								
Modified WFNS	≥2.5	0.604	82.7	77.7	3.702	0.223	0.839 (0.795-0.883)	<0.001
WFNS	≥3.5	0.589	75.9	83.0	4.461	0.289	0.837 (0.793-0.881)	<0.001
H&H	≥3.5	0.566	72.2	84.4	4.626	0.329	0.836 (0.791-0.881)	<0.001

Abbreviations: **AUC**, the area under the curve; **AUROC**, the area under the receiver operating characteristic; **CI**, confidence interval; **H&H**, Hunt and Hess scale; **LR**, likelihood ratio; **Modified WFNS**, Modified World Federation of Neurosurgical Societies scale; **SAH**, subarachnoid hemorrhage; **WFNS**, World Federation of Neurosurgical Societies scale.

S11 Table. Factors associated with poor outcome (mRS of 4 to 6) on day 30th after ictus in patients with aneurysmal subarachnoid haemorrhage (the exposure variable was defined as the originally-suggested 5-category modified WFNS grading scale)

Factors	Univariable logistic regression analyses[a]				Multivariable logistic regression analyses[b]			
	OR	95% CI for OR		p-value	AOR	95% CI for AOR		p-value
		Lower	Upper			Lower	Upper	
**Demographics**								
Age ≥ 60 years	2.407	1.586	3.654	<0.001	3.048	1.327	7.001	0.009

**Risk factors of aneurysmal SAH**								
Hypertension	2.056	1.354	3.122	0.001	NA	NA	NA	NA
**Comorbidities**								
Diabetes mellitus	3.751	1.669	8.433	0.001	NA	NA	NA	NA
**Neuroimaging findings on admission**								
Location of blood within the subarachnoid space:								
Basal cistern	2.685	1.728	4.173	<0.001	NA	NA	NA	NA
Sylvian fissure	2.981	1.125	7.900	0.028	NA	NA	NA	NA
Interhemispheric fissure	1.960	1.207	3.183	0.007	NA	NA	NA	NA
Interpeduncular fossa	3.254	1.995	5.308	<0.001	2.374	0.978	5.759	0.056
Suprasellar cistern	1.929	1.221	3.047	0.005	NA	NA	NA	NA
Ambient cistern	3.054	1.902	4.906	<0.001	NA	NA	NA	NA
Quadrigeminal cistern	5.141	3.270	8.085	<0.001	NA	NA	NA	NA
IVH	3.013	1.847	4.914	<0.001	NA	NA	NA	NA
ICH	1.860	1.142	3.029	0.013	NA	NA	NA	NA
Aneurysm locations								
PCoA aneurysm	0.554	0.299	1.026	0.061	NA	NA	NA	NA
VA aneurysm	3.341	1.265	8.820	0.015	NA	NA	NA	NA
**Severity of aneurysmal SAH on admission**								
Modified WFNS scale								
I	Reference			<0.001	Reference			<0.001
II	1.475	0.515	4.226	0.469	0.870	0.132	5.750	0.885
III	6.955	2.720	17.784	<0.001	6.475	1.481	28.313	0.013

IV	15.630	8.391	29.117	<0.001	9.550	3.254	28.033	<0.001
V	59.812	23.648	151.281	<0.001	20.515	4.671	90.112	<0.001
**Aneurysm repairs and other treatments**								
Aneurysm repairs:								
No aneurysm repair	Reference			<0.001	Reference			<0.001
Endovascular coiling	0.009	0.003	0.027	<0.001	0.010	0.002	0.040	<0.001
Surgical clipping	0.020	0.007	0.059	<0.001	0.013	0.003	0.055	<0.001
Surgical hematoma evacuation[c]	2.438	1.297	4.583	0.006	NA	NA	NA	NA
EVD[d]	3.537	1.846	6.777	<0.001	4.647	1.605	13.452	0.005
Nimodipine	0.076	0.028	0202	<0.001	NA	NA	NA	NA
**Complications**								
Rebleeding	7.868	2.537	24.396	<0.001	19.724	3.916	99.351	<0.001
DCI	7.316	2.848	18.792	<0.001	21.150	5.434	82.320	<0.001
Acute hydrocephalus	3.134	2.034	4.830	<0.001	NA	NA	NA	NA
Pneumonia	4.091	2.295	7.292	<0.001	2.772	1.074	7.155	0.035
Constant					0.664			0.612

[a]Each variable of the demographics, risk factors for aneurysmal SAH, comorbidities, initial clinical, neuroimaging and laboratory characteristics, the severity of aneurysmal SAH (i.e., modified WFNS scale) on admission, treatments, and complications was analyzed in the univariable logistic regression model and was considered in the multivariable logistic regression model if the P-value was <0.05 in univariable logistic regression analysis, as well as clinically crucial factors.

[b]All selected variables were included in the multivariable logistic regression model with the stepwise backward elimination method. Variables, then, were deleted stepwise from the full model until all remaining variables were independently associated with poor outcomes.

[c] Surgical hematoma evacuation was defined as any surgical procedure evacuating epidural, subdural, intraventricular, or intraparenchymal hematoma, such as decompressive craniotomy, open craniotomy, or minimally invasive surgery.

[d] The reason for the EVD insertion was the complication of acute hydrocephalus and others (See S5 and S9 Tables in S1 File for additional information).

Abbreviations: **AOR**, adjusted odds ratio; **CI**, confidence interval; **DCI**, delayed cerebral ischemia; **EVD**: external ventricular drainage; **ICH**, intracerebral hemorrhage; **IVH**, intraventricular hemorrhage; **mRS**, modified Rankin Scale; **NA**, not available; **OR**, odds ratio; **PCoA**, posterior communicating artery; **SAH**, subarachnoid hemorrhage; **VA**, vertebral artery; **WFNS**, World Federation of Neurosurgical Societies.

S12 Table. Factors associated with poor outcome (mRS of 4 to 6) on day 30th days ictus in patients with aneurysmal subarachnoid haemorrhage (the exposure variable was defined as the originally-suggested 5-category WFNS grading scale)

Factors	Univariable logistic regression analyses[a]				Multivariable logistic regression analyses[b]			
	OR	95% CI for OR		p-value	AOR	95% CI for AOR		p-value
		Lower	Upper			Lower	Upper	
**Demographics**								
Age ≥ 60 years	2.407	1.586	3.654	<0.001	2.971	1.296	6.809	0.010
**Risk factors of aneurysmal SAH**								
Hypertension	2.056	1.354	3.122	0.001	NA	NA	NA	NA
**Comorbidities**								
Diabetes mellitus	3.751	1.669	8.433	0.001	NA	NA	NA	NA
**Neuroimaging findings on admission**								
Location of blood within the subarachnoid space:								
Basal cistern	2.685	1.728	4.173	<0.001	NA	NA	NA	NA
Sylvian fissure	2.981	1.125	7.900	0.028	NA	NA	NA	NA
Interhemispheric fissure	1.960	1.207	3.183	0.007	NA	NA	NA	NA
Interpeduncular fossa	3.254	1.995	5.308	<0.001	2.361	0.972	5.737	0.058
Suprasellar cistern	1.929	1.221	3.047	0.005	NA	NA	NA	NA
Ambient cistern	3.054	1.902	4.906	<0.001	NA	NA	NA	NA
Quadrigeminal cistern	5.141	3.270	8.085	<0.001	NA	NA	NA	NA
IVH	3.013	1.847	4.914	<0.001	NA	NA	NA	NA
ICH	1.860	1.142	3.029	0.013	NA	NA	NA	NA
Aneurysm locations								
PCoA aneurysm	0.554	0.299	1.026	0.061	NA	NA	NA	NA

VA aneurysm	3.341	1.265	8.820	0.015	NA	NA	NA	NA
**Severity of aneurysmal SAH on admission**								
WFNS scale								
I	Reference			<0.001	Reference			<0.001
II	2.895	1.272	6.587	0.011	1.998	0.464	8.603	0.353
III	3.895	1.114	13.621	0.033	7.193	1.115	46.419	0.038
IV	15.630	8.391	29.117	<0.001	9.228	3.142	27.101	<0.001
V	59.812	23.648	151.281	<0.001	20.675	4.693	91.086	<0.001
**Aneurysm repairs and other treatments**								
Aneurysm repairs:								
No aneurysm repair	Reference			<0.001	Reference			<0.001
Endovascular coiling	0.009	0.003	0.027	<0.001	0.010	0.003	0.041	<0.001
Surgical clipping	0.020	0.007	0.059	<0.001	0.017	0.004	0.064	<0.001
Surgical hematoma evacuation[c]	2.438	1.297	4.583	0.006	NA	NA	NA	NA
EVD[d]	3.537	1.846	6.777	<0.001	5.153	1.743	15.238	0.003
Nimodipine	0.076	0.028	0202	<0.001	NA	NA	NA	NA
**Complications**								
Rebleeding	7.868	2.537	24.396	<0.001	17.457	3.360	90.709	0.001
DCI	7.316	2.848	18.792	<0.001	21.082	5.266	84.410	<0.001
Acute hydrocephalus	3.134	2.034	4.830	<0.001	NA	NA	NA	NA
Pneumonia	4.091	2.295	7.292	<0.001	3.004	1.141	7.911	0.023
Constant					0.582			0.497

[a]Each variable of the demographics, risk factors for aneurysmal SAH, comorbidities, initial clinical, neuroimaging and laboratory characteristics, the severity of aneurysmal SAH (i.e., modified WFNS scale) on admission, treatments, and complications was analyzed in the univariable logistic regression model and was considered in the multivariable logistic regression model if the P-value was <0.05 in univariable logistic regression analysis, as well as clinically crucial factors.

[b]All selected variables were included in the multivariable logistic regression model with the stepwise backward elimination method. Variables, then, were deleted stepwise from the full model until all remaining variables were independently associated with poor outcomes.

[c] Surgical hematoma evacuation was defined as any surgical procedure evacuating epidural, subdural, intraventricular, or intraparenchymal hematoma, such as decompressive craniotomy, open craniotomy, or minimally invasive surgery.

[d] The reason for the EVD insertion was the complication of acute hydrocephalus and others (See S5 and S9 Tables in S1 File for additional information).

Abbreviations: **AOR**, adjusted odds ratio; **CI**, confidence interval; **DCI**, delayed cerebral ischemia; **EVD**: external ventricular drainage; **ICH**, intracerebral hemorrhage; **IVH**, intraventricular hemorrhage; **mRS**, modified Rankin Scale; **NA**, not available; **OR**, odds ratio; **PCoA**, posterior communicating artery; **SAH**, subarachnoid hemorrhage; **VA**, vertebral artery; **WFNS**, World Federation of Neurosurgical Societies.

S13 Table. Factors associated with poor outcome (mRS of 4 to 6) on day 30th after ictus in patients with aneurysmal subarachnoid haemorrhage (the exposure variable was defined as the originally-suggested 5-category H&H grading scale)

Factors	Univariable logistic regression analyses[a]				Multivariable logistic regression analyses[b]			
	OR	95% CI for OR		p-value	AOR	95% CI for AOR		p-value
		Lower	Upper			Lower	Upper	
**Demographics**								
Age ≥ 60 years	2.407	1.586	3.654	<0.001	3.136	1.311	7.504	0.010
**Risk factors of aneurysmal SAH**								
Hypertension	2.056	1.354	3.122	0.001	NA	NA	NA	NA
**Comorbidities**								
Diabetes mellitus	3.751	1.669	8.433	0.001	NA	NA	NA	NA
**Neuroimaging findings on admission**								
Location of blood within the subarachnoid space:								
Basal cistern	2.685	1.728	4.173	<0.001	NA	NA	NA	NA
Sylvian fissure	2.981	1.125	7.900	0.028	NA	NA	NA	NA

Interhemispheric fissure	1.960	1.207	3.183	0.007	NA	NA	NA	NA
Interpeduncular fossa	3.254	1.995	5.308	<0.001	NA	NA	NA	NA
Suprasellar cistern	1.929	1.221	3.047	0.005	NA	NA	NA	NA
Ambient cistern	3.054	1.902	4.906	<0.001	NA	NA	NA	NA
Quadrigeminal cistern	5.141	3.270	8.085	<0.001	2.169	0.893	5.271	0.087
IVH	3.013	1.847	4.914	<0.001	NA	NA	NA	NA
ICH	1.860	1.142	3.029	0.013	NA	NA	NA	NA
Aneurysm locations								
PCoA aneurysm	0.554	0.299	1.026	0.061	NA	NA	NA	NA
VA aneurysm	3.341	1.265	8.820	0.015	NA	NA	NA	NA
**Severity of aneurysmal SAH on admission**								
H&H scale								
I	Reference			<0.001	Reference			<0.001
II	0.784	0.269	2.287	0.656	7.098	0.954	52.818	0.056
III	3.535	1.206	10.358	0.021	21.280	2.618	172.965	0.004
IV	7.360	2.478	21.860	<0.001	25.988	3.271	206.506	0.002
V	38.000	12.973	111.306	<0.001	102.383	12.828	817.115	<0.001
**Aneurysm repairs and other treatments**								
Aneurysm repairs:								
No aneurysm repair	Reference			<0.001	Reference			<0.001
Endovascular coiling	0.009	0.003	0.027	<0.001	0.007	0.002	0.031	<0.001
Surgical clipping	0.020	0.007	0.059	<0.001	0.012	0.003	0.051	<0.001
Surgical hematoma evacuation[c]	2.438	1.297	4.583	0.006	NA	NA	NA	NA
EVD[d]	3.537	1.846	6.777	<0.001	5.085	1.743	14.833	0.003

Nimodipine	0.076	0.028	0202	<0.001	NA	NA	NA	NA
**Complications**								
Rebleeding	7.868	2.537	24.396	<0.001	22.967	4.516	116.793	<0.001
DCI	7.316	2.848	18.792	<0.001	63.837	10.127	402.406	<0.001
Acute hydrocephalus	3.134	2.034	4.830	<0.001	NA	NA	NA	NA
Pneumonia	4.091	2.295	7.292	<0.001	3.504	1.296	9.474	0.013
Constant					0.205			0.132

[a]Each variable of the demographics, risk factors for aneurysmal SAH, comorbidities, initial clinical, neuroimaging and laboratory characteristics, the severity of aneurysmal SAH (i.e., H&H scale) on admission, treatments, and complications was analyzed in the univariable logistic regression model and was considered in the multivariable logistic regression model if the P-value was <0.05 in univariable logistic regression analysis, as well as clinically crucial factors.

[b]All selected variables were included in the multivariable logistic regression model with the stepwise backward elimination method. Variables, then, were deleted stepwise from the full model until all remaining variables were independently associated with poor outcomes.

[c] Surgical hematoma evacuation was defined as any surgical procedure evacuating epidural, subdural, intraventricular, or intraparenchymal hematoma, such as decompressive craniotomy, open craniotomy, or minimally invasive surgery.

[d] The reason for the EVD insertion was the complication of acute hydrocephalus and others (See S5 and S9 Tables in S1 File for additional information).

Abbreviations: **AOR**, adjusted odds ratio; **CI**, confidence interval; **DCI**, delayed cerebral ischemia; **EVD**: external ventricular drainage; **H&H**, Hunt and Hess; **ICH**, intracerebral hemorrhage; **IVH**, intraventricular hemorrhage; **mRS**, modified Rankin Scale; **NA**, not available; **OR**, odds ratio; **PCoA**, posterior communicating artery; **SAH**, subarachnoid hemorrhage; **VA**, vertebral artery.

S14 Table. Factors associated with poor outcome (mRS of 4 to 6) on day 90th after ictus in patients with aneurysmal subarachnoid haemorrhage (the exposure variable was defined as the originally-suggested 5-category modified WFNS grading scale)

Factors	Univariable logistic regression analyses[a]				Multivariable logistic regression analyses[b]			
	OR	95% CI for OR		p-value	AOR	95% CI for AOR		p-value
		Lower	Upper			Lower	Upper	
**Demographics**								
Age ≥ 60 years	2.581	1.691	3.939	<0.001	4.144	1.719	9.988	0.002
**Risk factors of aneurysmal SAH**								

Hypertension	2.133	1.400	3.250	<0.001	NA	NA	NA	NA
**Comorbidities**								
Diabetes mellitus	3.986	1.772	8.968	0.001	NA	NA	NA	NA
**Neuroimaging findings on admission**								
Location of blood within the subarachnoid space:								
Basal cistern	2.718	1.738	4.251	<0.001	NA	NA	NA	NA
Sylvian fissure	2.811	1.060	7.454	0.038	NA	NA	NA	NA
Interhemispheric fissure	2.063	1.257	3.385	0.004	NA	NA	NA	NA
Interpeduncular fossa	3.206	1.953	5.264	<0.001	NA	NA	NA	NA
Suprasellar cistern	1.882	1.186	2.986	0.007	NA	NA	NA	NA
Ambient cistern	2.990	1.852	4.829	<0.001	NA	NA	NA	NA
Quadrigeminal cistern	4.414	3.431	8.545	<0.001	NA	NA	NA	NA
IVH	2.793	1.711	4.559	<0.001	NA	NA	NA	NA
ICH	1.879	1.151	3.068	0.012	NA	NA	NA	NA
Aneurysm locations								
PCoA aneurysm	0.590	0.318	1.094	0.094	NA	NA	NA	NA
VA aneurysm	3.542	1.341	9.356	0.011	NA	NA	NA	NA
**Severity of aneurysmal SAH on admission**								
Modified WFNS scale								
I	Reference			<0.001	Reference			<0.001
II	1.566	0.544	4.509	0.406	0.990	0.140	6.977	0.992
III	6.200	2.380	16.154	<0.001	6.242	1.354	28.762	0.019
IV	14.618	7.803	27.383	<0.001	7.110	2.285	22.118	0.001
V	63.476	24.947	161.511	<0.001	22.720	4.921	104.901	<0.001

**Aneurysm repairs and other treatments**								
Aneurysm repairs:								
No aneurysm repair	Reference			<0.001	Reference			<0.001
Endovascular coiling	0.011	0.004	0.030	<0.001	0.009	0.002	0.035	<0.001
Surgical clipping	0.023	0.009	0.062	<0.001	0.010	0.002	0.044	<0.001
Surgical hematoma evacuation[c]	2.111	1.123	3.971	0.020	NA	NA	NA	NA
EVD[d]	3.382	1.773	6.453	<0.001	4.128	1.282	13.289	0.017
Nimodipine	0.071	0.027	0.191	<0.001	NA	NA	NA	NA
**Complications**								
Rebleeding	6.133	2.138	17.594	0.001	18.751	3.224	109.042	0.001
DCI	6.245	2.538	15.365	<0.001	21.478	5.354	86.159	<0.001
Acute hydrocephalus	3.134	2.028	4.842	<0.001	2.276	0.888	5.834	0.087
Pneumonia	4.022	2.263	7.148	<0.001	2.725	1.010	7.355	0.048
Constant					0.703			0.637

[a]Each variable of the demographics, risk factors for aneurysmal SAH, comorbidities, initial clinical, neuroimaging and laboratory characteristics, the severity of aneurysmal SAH (i.e., modified WFNS scale) on admission, treatments, and complications was analyzed in the univariable logistic regression model and was considered in the multivariable logistic regression model if the P-value was <0.05 in univariable logistic regression analysis, as well as clinically crucial factors.

[b]All selected variables were included in the multivariable logistic regression model with the stepwise backward elimination method. Variables, then, were deleted stepwise from the full model until all remaining variables were independently associated with poor outcomes.

[c] Surgical hematoma evacuation was defined as any surgical procedure evacuating epidural, subdural, intraventricular, or intraparenchymal hematoma, such as decompressive craniotomy, open craniotomy, or minimally invasive surgery.

[d] The reason for the EVD insertion was the complication of acute hydrocephalus and others (See S5 and S9 Tables in S1 File for additional information).

Abbreviations: **AOR**, adjusted odds ratio; **CI**, confidence interval; **DCI**, delayed cerebral ischemia; **EVD**: external ventricular drainage; **ICH**, intracerebral hemorrhage; **IVH**, intraventricular hemorrhage; **mRS**, modified Rankin Scale; **NA**, not available; **OR**, odds ratio; **PCoA**, posterior communicating artery; **SAH**, subarachnoid hemorrhage; **VA**, vertebral artery; **WFNS**, World Federation of Neurosurgical Societies.

S15 Table. Factors associated with poor outcome (mRS of 4 to 6) on day 90th after ictus in patients with aneurysmal subarachnoid haemorrhage (the exposure variable was defined as the originally-suggested 5-category WFNS grading scale)

Factors	Univariable logistic regression analyses[a]				Multivariable logistic regression analyses[b]			
	OR	95% CI for OR		p-value	AOR	95% CI for AOR		p-value
		Lower	Upper			Lower	Upper	
**Demographics**								
Age ≥ 60 years	2.581	1.691	3.939	<0.001	3.881	1.646	9.151	0.002
**Risk factors of aneurysmal SAH**								
Hypertension	2.133	1.400	3.250	<0.001	NA	NA	NA	NA
**Comorbidities**								
Diabetes mellitus	3.986	1.772	8.968	0.001	NA	NA	NA	NA
**Neuroimaging findings on admission**								
Location of blood within the subarachnoid space:								
Basal cistern	2.718	1.738	4.251	<0.001	NA	NA	NA	NA
Sylvian fissure	2.811	1.060	7.454	0.038	NA	NA	NA	NA
Interhemispheric fissure	2.063	1.257	3.385	0.004	NA	NA	NA	NA
Interpeduncular fossa	3.206	1.953	5.264	<0.001	NA	NA	NA	NA
Suprasellar cistern	1.882	1.186	2.986	0.007	NA	NA	NA	NA
Ambient cistern	2.990	1.852	4.829	<0.001	NA	NA	NA	NA
Quadrigeminal cistern	4.414	3.431	8.545	<0.001	NA	NA	NA	NA
IVH	2.793	1.711	4.559	<0.001	NA	NA	NA	NA
ICH	1.879	1.151	3.068	0.012	NA	NA	NA	NA
Aneurysm locations								
PCoA aneurysm	0.590	0.318	1.094	0.094	NA	NA	NA	NA

VA aneurysm	3.542	1.341	9.356	0.011	NA	NA	NA	NA
**Severity of aneurysmal SAH on admission**								
WFNS score of 4 to 5								
I	Reference			<0.001	Reference			<0.001
II	2.719	1.164	6.350	0.021	2.097	0.493	8.914	0.316
III	4.133	1.177	14.520	0.027	6.198	0.946	40.582	0.057
IV	14.618	7.803	27.383	<0.001	9.132	3.090	26.989	<0.001
V	63.476	24.947	161.511	<0.001	27.464	6.145	122.742	<0.001
**Aneurysm repairs and other treatments**								
Aneurysm repairs:								
No aneurysm repair	Reference			<0.001	Reference			<0.001
Endovascular coiling	0.011	0.004	0.030	<0.001	0.011	0.003	0.039	<0.001
Surgical clipping	0.023	0.009	0.062	<0.001	0.019	0.005	0.067	<0.001
Surgical hematoma evacuation[c]	2.111	1.123	3.971	0.020	NA	NA	NA	NA
EVD[d]	3.382	1.773	6.453	<0.001	6.413	2.085	19.725	0.001
Nimodipine	0.071	0.027	0.191	<0.001	NA	NA	NA	NA
**Complications**								
Rebleeding	6.133	2.138	17.594	0.001	20.101	3.583	112.780	0.001
DCI	6.245	2.538	15.365	<0.001	16.8387	4.380	64.739	<0.001
Acute hydrocephalus	3.134	2.028	4.842	<0.001	NA	NA	NA	NA
Pneumonia	4.022	2.263	7.148	<0.001	3.671	1.375	9.803	0.009
Constant					0.675			0.582

[a]Each variable of the demographics, risk factors for aneurysmal SAH, comorbidities, initial clinical, neuroimaging and laboratory characteristics, the severity of aneurysmal SAH (i.e., modified WFNS scale) on admission, treatments, and complications was analyzed in the univariable logistic regression model and was considered in the multivariable logistic regression model if the P-value was <0.05 in univariable logistic regression analysis, as well as clinically crucial factors.

[b]All selected variables were included in the multivariable logistic regression model with the stepwise backward elimination method. Variables, then, were deleted stepwise from the full model until all remaining variables were independently associated with poor outcomes.

[c] Surgical hematoma evacuation was defined as any surgical procedure evacuating epidural, subdural, intraventricular, or intraparenchymal hematoma, such as decompressive craniotomy, open craniotomy, or minimally invasive surgery.

[d] The reason for the EVD insertion was the complication of acute hydrocephalus and others (See S5 and S9 Tables in S1 File for additional information).

Abbreviations: **AOR**, adjusted odds ratio; **CI**, confidence interval; **DCI**, delayed cerebral ischemia; **EVD**: external ventricular drainage; **ICH**, intracerebral hemorrhage; **IVH**, intraventricular hemorrhage; **mRS**, modified Rankin Scale; **NA**, not available; **OR**, odds ratio; **PCoA**, posterior communicating artery; **SAH**, subarachnoid hemorrhage; **VA**, vertebral artery; **WFNS**, World Federation of Neurosurgical Societies.

S16 Table. Factors associated with poor outcome (mRS of 4 to 6) on day 90th after ictus in patients with aneurysmal subarachnoid haemorrhage (the exposure variable was defined as the originally-suggested 5-category H&H grading scale)

Factors	Univariable logistic regression analyses[a]				Multivariable logistic regression analyses[b]			
	OR	95% CI for OR		p-value	AOR	95% CI for AOR		p-value
		Lower	Upper			Lower	Upper	
**Demographics**								
Age ≥ 60 years	2.581	1.691	3.939	<0.001	4.162	1.680	10.310	0.002
**Risk factors of aneurysmal SAH**								
Hypertension	2.133	1.400	3.250	<0.001	NA	NA	NA	NA
**Comorbidities**								
Diabetes mellitus	3.986	1.772	8.968	0.001	4.252	0.863	20.956	0.075
**Neuroimaging findings on admission**								
Location of blood within the subarachnoid space:								
Basal cistern	2.718	1.738	4.251	<0.001	NA	NA	NA	NA
Sylvian fissure	2.811	1.060	7.454	0.038	NA	NA	NA	NA

Interhemispheric fissure	2.063	1.257	3.385	0.004	NA	NA	NA	NA
Interpeduncular fossa	3.206	1.953	5.264	<0.001	NA	NA	NA	NA
Suprasellar cistern	1.882	1.186	2.986	0.007	NA	NA	NA	NA
Ambient cistern	2.990	1.852	4.829	<0.001	NA	NA	NA	NA
Quadrigeminal cistern	4.414	3.431	8.545	<0.001	2.306	0.949	5.605	0.065
IVH	2.793	1.711	4.559	<0.001	NA	NA	NA	NA
ICH	1.879	1.151	3.068	0.012	NA	NA	NA	NA
Aneurysm locations								
PCoA aneurysm	0.590	0.318	1.094	0.094	NA	NA	NA	NA
VA aneurysm	3.542	1.341	9.356	0.011	NA	NA	NA	NA
**Severity of aneurysmal SAH on admission**								
H&H scale								
I	Reference			<0.001	Reference			<0.001
II	0.727	0.247	2.139	0.563	4.574	0.683	30.654	0.117
III	3.273	1.112	9.631	0.031	14.762	2.029	107.420	0.008
IV	6.769	2.277	20.121	0.001	17.161	2.412	122.125	0.005
V	32.889	11.362	95.201	<0.001	75.501	10.497	543.035	<0.001
**Aneurysm repairs and other treatments**								
Aneurysm repairs:								
No aneurysm repair	Reference			<0.001	Reference			<0.001
Endovascular coiling	0.011	0.004	0.030	<0.001	0.008	0.002	0.035	<0.001
Surgical clipping	0.023	0.009	0.062	<0.001	0.014	0.003	0.055	<0.001
Surgical hematoma evacuation[c]	2.111	1.123	3.971	0.020	NA	NA	NA	NA
EVD[d]	3.382	1.773	6.453	<0.001	6.999	2.286	21.431	0.001

Nimodipine	0.071	0.027	0.191	<0.001	NA	NA	NA	NA
**Complications**								
Rebleeding	6.133	2.138	17.594	0.001	24.523	4.372	137.567	<0.001
DCI	6.245	2.538	15.365	<0.001	35.196	6.767	183.055	<0.001
Acute hydrocephalus	3.134	2.028	4.842	<0.001	NA	NA	NA	NA
Pneumonia	4.022	2.263	7.148	<0.001	3.455	1.234	9.679	0.018
Constant					0.163			0.074

[a]Each variable of the demographics, risk factors for aneurysmal SAH, comorbidities, initial clinical, neuroimaging and laboratory characteristics, the severity of aneurysmal SAH (i.e., H&H scale) on admission, treatments, and complications was analyzed in the univariable logistic regression model and was considered in the multivariable logistic regression model if the P-value was <0.05 in univariable logistic regression analysis, as well as clinically crucial factors.

[b]All selected variables were included in the multivariable logistic regression model with the stepwise backward elimination method. Variables, then, were deleted stepwise from the full model until all remaining variables were independently associated with poor outcomes.

[c] Surgical hematoma evacuation was defined as any surgical procedure evacuating epidural, subdural, intraventricular, or intraparenchymal hematoma, such as decompressive craniotomy, open craniotomy, or minimally invasive surgery.

[d] The reason for the EVD insertion was the complication of acute hydrocephalus and others (See S5 and S9 Tables in S1 File for additional information).

Abbreviations: **AOR**, adjusted odds ratio; **CI**, confidence interval; **DCI**, delayed cerebral ischemia; **EVD**: external ventricular drainage; **H&H**, Hunt and Hess; **ICH**, intracerebral hemorrhage; **IVH**, intraventricular hemorrhage; **mRS**, modified Rankin Scale; **NA**, not available; **OR**, odds ratio; **PCoA**, posterior communicating artery; **SAH**, subarachnoid hemorrhage; **VA**, vertebral artery.

S17 Table. Factors associated with poor outcome (mRS of 4 to 6) on day 30th after ictus in patients with aneurysmal subarachnoid haemorrhage (the exposure variable was defined as the grades of the WFNS scale which were higher than or equal to the cut-off value)

Factors	Univariable logistic regression analyses[a]				Multivariable logistic regression analyses[b]			
	OR	95% CI for OR		p-value	AOR	95% CI for AOR		p-value
		Lower	Upper			Lower	Upper	
**Demographics**								
Age ≥ 60 years	2.407	1.586	3.654	<0.001	2.712	1.204	6.109	0.016
**Risk factors of aneurysmal SAH**								

Hypertension	2.056	1.354	3.122	0.001	NA	NA	NA	NA
**Comorbidities**								
Diabetes mellitus	3.751	1.669	8.433	0.001	NA	NA	NA	NA
**Neuroimaging findings on admission**								
Location of blood within the subarachnoid space:								
Basal cistern	2.685	1.728	4.173	<0.001	NA	NA	NA	NA
Sylvian fissure	2.981	1.125	7.900	0.028	NA	NA	NA	NA
Interhemispheric fissure	1.960	1.207	3.183	0.007	NA	NA	NA	NA
Interpeduncular fossa	3.254	1.995	5.308	<0.001	NA	NA	NA	NA
Suprasellar cistern	1.929	1.221	3.047	0.005	NA	NA	NA	NA
Ambient cistern	3.054	1.902	4.906	<0.001	NA	NA	NA	NA
Quadrigeminal cistern	5.141	3.270	8.085	<0.001	2.295	0.990	5.321	0.053
IVH	3.013	1.847	4.914	<0.001	NA	NA	NA	NA
ICH	1.860	1.142	3.029	0.013	NA	NA	NA	NA
Aneurysm locations								
PCoA aneurysm	0.554	0.299	1.026	0.061	NA	NA	NA	NA
VA aneurysm	3.341	1.265	8.820	0.015	NA	NA	NA	NA
**Severity of aneurysmal SAH on admission**								
WFNS grade of IV to V[c]	15.770	9.547	26.048	<0.001	6.879	2.884	16.408	<0.001
**Aneurysm repairs and other treatments**								
Aneurysm repairs:								
No aneurysm repair	Reference			<0.001	Reference			<0.001
Endovascular coiling	0.009	0.003	0.027	<0.001	0.008	0.002	0.031	<0.001

Surgical clipping	0.020	0.007	0.059	<0.001	0.015	0.004	0.056	<0.001
Surgical hematoma evacuation[d]	2.438	1.297	4.583	0.006	NA	NA	NA	NA
EVD[e]	3.537	1.846	6.777	<0.001	4.761	1.672	13.553	0.003
Nimodipine	0.076	0.028	0202	<0.001	NA	NA	NA	NA
**Complications**						1.672	13.553	
Rebleeding	7.868	2.537	24.396	<0.001	18.084	3.852	84.908	<0.001
DCI	7.316	2.848	18.792	<0.001	17.574	4.635	66.640	<0.001
Acute hydrocephalus	3.134	2.034	4.830	<0.001	NA	NA	NA	NA
Pneumonia	4.091	2.295	7.292	<0.001	3.553	1.371	9.206	0.009
Constant					1.409			0.598

[a]Each variable of the demographics, risk factors for aneurysmal SAH, comorbidities, initial clinical, neuroimaging and laboratory characteristics, the severity of aneurysmal SAH (i.e., modified WFNS scale) on admission, treatments, and complications was analyzed in the univariable logistic regression model and was considered in the multivariable logistic regression model if the P-value was <0.05 in univariable logistic regression analysis, as well as clinically crucial factors.

[b]All selected variables were included in the multivariable logistic regression model with the stepwise backward elimination method. Variables, then, were deleted stepwise from the full model until all remaining variables were independently associated with poor outcomes.

[c] The grades of the WFNS scale which were higher than or equal to the cut-off value.

[d] Surgical hematoma evacuation was defined as any surgical procedure evacuating epidural, subdural, intraventricular, or intraparenchymal hematoma, such as decompressive craniotomy, open craniotomy, or minimally invasive surgery.

[e] The reason for the EVD insertion was the complication of acute hydrocephalus and others (See S5 and S9 Tables in S1 File for additional information).

Abbreviations: **AOR**, adjusted odds ratio; **CI**, confidence interval; **DCI**, delayed cerebral ischemia; **EVD**: external ventricular drainage; **ICH**, intracerebral hemorrhage; **IVH**, intraventricular hemorrhage; **mRS**, modified Rankin Scale; **NA**, not available; **OR**, odds ratio; **PCoA**, posterior communicating artery; **SAH**, subarachnoid hemorrhage; **VA**, vertebral artery; **WFNS**, World Federation of Neurosurgical Societies.

S18 Table. Factors associated with poor outcome (mRS of 4 to 6) on day 30th after ictus in patients with aneurysmal subarachnoid haemorrhage (the exposure variable was defined as the grades of the H&H scale which were higher than or equal to the cut-off value)

Factors	Univariable logistic regression analyses[a]				Multivariable logistic regression analyses[b]			
	OR	95% CI for OR		p-value	AOR	95% CI for AOR		p-value
		Lower	Upper			Lower	Upper	
**Demographics**								
Age ≥ 60 years	2.407	1.586	3.654	<0.001	3.240	1.424	7.372	0.005
**Risk factors of aneurysmal SAH**								
Hypertension	2.056	1.354	3.122	0.001	NA	NA	NA	NA
**Comorbidities**								
Diabetes mellitus	3.751	1.669	8.433	0.001	NA	NA	NA	NA
**Neuroimaging findings on admission**								
Location of blood within the subarachnoid space:								
Basal cistern	2.685	1.728	4.173	<0.001	NA	NA	NA	NA
Sylvian fissure	2.981	1.125	7.900	0.028	NA	NA	NA	NA
Interhemispheric fissure	1.960	1.207	3.183	0.007	NA	NA	NA	NA
Interpeduncular fossa	3.254	1.995	5.308	<0.001	2.508	1.044	6.023	0.040
Suprasellar cistern	1.929	1.221	3.047	0.005	NA	NA	NA	NA
Ambient cistern	3.054	1.902	4.906	<0.001	NA	NA	NA	NA
Quadrigeminal cistern	5.141	3.270	8.085	<0.001	NA	NA	NA	NA
IVH	3.013	1.847	4.914	<0.001	NA	NA	NA	NA
ICH	1.860	1.142	3.029	0.013	NA	NA	NA	NA
Aneurysm locations								
PCoA aneurysm	0.554	0.299	1.026	0.061	NA	NA	NA	NA

VA aneurysm	3.341	1.265	8.820	0.015	NA	NA	NA	NA
**Severity of aneurysmal SAH on admission**								
H&H grade of IV to V[c]	14.612	8.887	24.025	<0.001	7.475	3.202	17.449	<0.001
**Aneurysm repairs and other treatments**								
Aneurysm repairs:								
No aneurysm repair	Reference			<0.001	Reference			<0.001
Endovascular coiling	0.009	0.003	0.027	<0.001	0.009	0.002	0.032	<0.001
Surgical clipping	0.020	0.007	0.059	<0.001	0.013	0.003	0.046	<0.001
Surgical hematoma evacuation[d]	2.438	1.297	4.583	0.006	NA	NA	NA	NA
EVD[e]	3.537	1.846	6.777	<0.001	4.839	1.658	14.125	0.004
Nimodipine	0.076	0.028	0202	<0.001	NA	NA	NA	NA
**Complications**								
Rebleeding	7.868	2.537	24.396	<0.001	18.916	4.055	88.246	<0.001
DCI	7.316	2.848	18.792	<0.001	15.129	3.927	58.295	<0.001
Acute hydrocephalus	3.134	2.034	4.830	<0.001	NA	NA	NA	NA
Pneumonia	4.091	2.295	7.292	<0.001	3.981	1.546	10.255	0.004
Constant					1.031			0.965

[a]Each variable of the demographics, risk factors for aneurysmal SAH, comorbidities, initial clinical, neuroimaging and laboratory characteristics, the severity of aneurysmal SAH (i.e., H&H scale) on admission, treatments, and complications was analyzed in the univariable logistic regression model and was considered in the multivariable logistic regression model if the P-value was <0.05 in univariable logistic regression analysis, as well as clinically crucial factors.

[b]All selected variables were included in the multivariable logistic regression model with the stepwise backward elimination method. Variables, then, were deleted stepwise from the full model until all remaining variables were independently associated with poor outcomes.

[c] The grades of the H&H scale which were higher than or equal to the cut-off value.

[d] Surgical hematoma evacuation was defined as any surgical procedure evacuating epidural, subdural, intraventricular, or intraparenchymal hematoma, such as decompressive craniotomy, open craniotomy, or minimally invasive surgery.

[e] The reason for the EVD insertion was the complication of acute hydrocephalus and others (See S5 and S9 Tables in S1 File for additional information).

Abbreviations: **AOR**, adjusted odds ratio; **CI**, confidence interval; **DCI**, delayed cerebral ischemia; **EVD**: external ventricular drainage; **H&H**, Hunt and Hess; **ICH**, intracerebral hemorrhage; **IVH**, intraventricular hemorrhage; **mRS**, modified Rankin Scale; **NA**, not available; **OR**, odds ratio; **PCoA**, posterior communicating artery; **SAH**, subarachnoid hemorrhage; **VA**, vertebral artery.

S19 Table. Factors associated with poor outcome (mRS of 4 to 6) on day 90th after ictus in patients with aneurysmal subarachnoid haemorrhage (the exposure variable was defined as the grades of the WFNS scale which were higher than or equal to the cut-off value)

Factors	Univariable logistic regression analyses[a]				Multivariable logistic regression analyses[b]			
	OR	95% CI for OR		p-value	AOR	95% CI for AOR		p-value
		Lower	Upper			Lower	Upper	
**Demographics**								
Age ≥ 60 years	2.581	1.691	3.939	<0.001	3.502	1.521	8.062	0.003
**Risk factors of aneurysmal SAH**								
Hypertension	2.133	1.400	3.250	<0.001	NA	NA	NA	NA
**Comorbidities**								
Diabetes mellitus	3.986	1.772	8.968	0.001	NA	NA	NA	NA
**Neuroimaging findings on admission**								
Location of blood within the subarachnoid space:								
Basal cistern	2.718	1.738	4.251	<0.001	NA	NA	NA	NA
Sylvian fissure	2.811	1.060	7.454	0.038	NA	NA	NA	NA
Interhemispheric fissure	2.063	1.257	3.385	0.004	NA	NA	NA	NA
Interpeduncular fossa	3.206	1.953	5.264	<0.001	NA	NA	NA	NA
Suprasellar cistern	1.882	1.186	2.986	0.007	NA	NA	NA	NA
Ambient cistern	2.990	1.852	4.829	<0.001	NA	NA	NA	NA

Quadrigeminal cistern	4.414	3.431	8.545	<0.001	2.380	1.024	5.534	0.044
IVH	2.793	1.711	4.559	<0.001	NA	NA	NA	NA
ICH	1.879	1.151	3.068	0.012	NA	NA	NA	NA
Aneurysm locations								
PCoA aneurysm	0.590	0.318	1.094	0.094	NA	NA	NA	NA
VA aneurysm	3.542	1.341	9.356	0.011	NA	NA	NA	NA
**Severity of aneurysmal SAH on admission**								
WFNS grade of IV to V[c]	15.387	9.291	25.483	<0.001	6.383	2.661	15.310	<0.001
**Aneurysm repairs and other treatments**								
Aneurysm repairs:								
No aneurysm repair	Reference			<0.001	Reference			<0.001
Endovascular coiling	0.011	0.004	0.030	<0.001	0.008	0.002	0.030	<0.001
Surgical clipping	0.023	0.009	0.062	<0.001	0.017	0.005	0.058	<0.001
Surgical hematoma evacuation[d]	2.111	1.123	3.971	0.020	NA	NA	NA	NA
EVD[e]	3.382	1.773	6.453	<0.001	5.489	1.891	15.934	0.002
Nimodipine	0.071	0.027	0.191	<0.001	NA	NA	NA	NA
**Complications**								
Rebleeding	6.133	2.138	17.594	0.001	19.498	4.102	92.683	<0.001
DCI	6.245	2.538	15.365	<0.001	12.899	3.484	47.762	<0.001
Acute hydrocephalus	3.134	2.028	4.842	<0.001	NA	NA	NA	NA
Pneumonia	4.022	2.263	7.148	<0.001	4.116	1.573	10.769	0.004
Constant					1.035			0.955

[a]Each variable of the demographics, risk factors for aneurysmal SAH, comorbidities, initial clinical, neuroimaging and laboratory characteristics, the severity of aneurysmal SAH (i.e., modified WFNS scale) on admission, treatments, and complications was analyzed in the univariable logistic regression model and was considered in the multivariable logistic regression model if the P-value was <0.05 in univariable logistic regression analysis, as well as clinically crucial factors.

[b]All selected variables were included in the multivariable logistic regression model with the stepwise backward elimination method. Variables, then, were deleted stepwise from the full model until all remaining variables were independently associated with poor outcomes.

[c] The grades of the WFNS scale which were higher than or equal to the cut-off value.

[d] Surgical hematoma evacuation was defined as any surgical procedure evacuating epidural, subdural, intraventricular, or intraparenchymal hematoma, such as decompressive craniotomy, open craniotomy, or minimally invasive surgery.

[e] The reason for the EVD insertion was the complication of acute hydrocephalus and others (See S5 and S9 Tables in S1 File for additional information).

Abbreviations: **AOR**, adjusted odds ratio; **CI**, confidence interval; **DCI**, delayed cerebral ischemia; **EVD**: external ventricular drainage; **ICH**, intracerebral hemorrhage; **IVH**, intraventricular hemorrhage; **mRS**, modified Rankin Scale; **NA**, not available; **OR**, odds ratio; **PCoA**, posterior communicating artery; **SAH**, subarachnoid hemorrhage; **VA**, vertebral artery; **WFNS**, World Federation of Neurosurgical Societies.

S20 Table. Factors associated with poor outcome (mRS of 4 to 6) on day 90th after ictus in patients with aneurysmal subarachnoid haemorrhage (the exposure variable was defined as the grades of the H&H scale which were higher than or equal to the cut-off value)

Factors	Univariable logistic regression analyses[a]				Multivariable logistic regression analyses[b]			
	OR	95% CI for OR		p-value	AOR	95% CI for AOR		p-value
		Lower	Upper			Lower	Upper	
**Demographics**								
Age ≥ 60 years	2.581	1.691	3.939	<0.001	3.856	1.659	8.963	0.002
**Risk factors of aneurysmal SAH**								
Hypertension	2.133	1.400	3.250	<0.001	NA	NA	NA	NA
**Comorbidities**								
Diabetes mellitus	3.986	1.772	8.968	0.001	NA	NA	NA	NA
**Neuroimaging findings on admission**								
Location of blood within the subarachnoid space:								
Basal cistern	2.718	1.738	4.251	<0.001	NA	NA	NA	NA

Sylvian fissure	2.811	1.060	7.454	0.038	NA	NA	NA	NA
Interhemispheric fissure	2.063	1.257	3.385	0.004	NA	NA	NA	NA
Interpeduncular fossa	3.206	1.953	5.264	<0.001	NA	NA	NA	NA
Suprasellar cistern	1.882	1.186	2.986	0.007	NA	NA	NA	NA
Ambient cistern	2.990	1.852	4.829	<0.001	NA	NA	NA	NA
Quadrigeminal cistern	4.414	3.431	8.545	<0.001	2.542	1.090	5.925	0.031
IVH	2.793	1.711	4.559	<0.001	NA	NA	NA	NA
ICH	1.879	1.151	3.068	0.012	NA	NA	NA	NA
Aneurysm locations								
PCoA aneurysm	0.590	0.318	1.094	0.094	NA	NA	NA	NA
VA aneurysm	3.542	1.341	9.356	0.011	NA	NA	NA	NA
**Severity of aneurysmal SAH on admission**								
H&H grade of IV to V[c]	14.034	8.535	23.076	<0.001	6.146	2.584	14.620	<0.001
**Aneurysm repairs and other treatments**								
Aneurysm repairs:								
No aneurysm repair	Reference			<0.001	Reference			<0.001
Endovascular coiling	0.011	0.004	0.030	<0.001	0.009	0.002	0.031	<0.001
Surgical clipping	0.023	0.009	0.062	<0.001	0.016	0.005	0.055	<0.001
Surgical hematoma evacuation[d]	2.111	1.123	3.971	0.020	NA	NA	NA	NA
EVD[e]	3.382	1.773	6.453	<0.001	5.632	1.932	16.413	0.002
Nimodipine	0.071	0.027	0.191	<0.001	NA	NA	NA	NA
**Complications**								
Rebleeding	6.133	2.138	17.594	0.001	22.161	4.881	100.609	<0.001
DCI	6.245	2.538	15.365	<0.001	12.087	3.294	44.352	<0.001

Acute hydrocephalus	3.134	2.028	4.842	<0.001	NA	NA	NA	NA
Pneumonia	4.022	2.263	7.148	<0.001	4.971	1.884	13.115	0.001
Constant					1.059			0.926

[a]Each variable of the demographics, risk factors for aneurysmal SAH, comorbidities, initial clinical, neuroimaging and laboratory characteristics, the severity of aneurysmal SAH (i.e., H&H scale) on admission, treatments, and complications was analyzed in the univariable logistic regression model and was considered in the multivariable logistic regression model if the P-value was <0.05 in univariable logistic regression analysis, as well as clinically crucial factors.

[b]All selected variables were included in the multivariable logistic regression model with the stepwise backward elimination method. Variables, then, were deleted stepwise from the full model until all remaining variables were independently associated with poor outcomes.

[c] The grades of the H&H scale which were higher than or equal to the cut-off value.

[d] Surgical hematoma evacuation was defined as any surgical procedure evacuating epidural, subdural, intraventricular, or intraparenchymal hematoma, such as decompressive craniotomy, open craniotomy, or minimally invasive surgery.

[e] The reason for the EVD insertion was the complication of acute hydrocephalus and others (See S5 and S9 Tables in S1 File for additional information).

Abbreviations: **AOR**, adjusted odds ratio; **CI**, confidence interval; **DCI**, delayed cerebral ischemia; **H&H**, Hunt and Hess; **ICH**, intracerebral hemorrhage; **IVH**, intraventricular hemorrhage; **mRS**, modified Rankin Scale; **NA**, not available; **OR**, odds ratio; **PCoA**, posterior communicating artery; **SAH**, subarachnoid hemorrhage; **VA**, vertebral artery.

S21 Table. Breakdown of missing data

Variables	Number of patients with missing data
Prehospital setting	
Transferred from local hospitals	0
Hospital taken to	
Viet Duc	0
Bach Mai	0
Hanoi Medical University	0
**Demographics**	
Age (year)	0
Gender (male)	0
**Risk factors of aneurysmal SAH**	
Cigarette smoking	0
Hypertension	2
Genetic risk	0
Alcohol consumption	14
Sympathomimetic drugs	2
Oestrogen deficiency	4
Antithrombotic therapy	0
Elevated total cholesterol	0
**Comorbidities**	
Cerebrovascular disease	0
Chronic cardiac failure	0
Coronary artery disease/MI	0
COPD/Asthma	0
Active neoplasm	0
Chronic renal failure	0
Ulcer disease	0
Diabetes mellitus	0
Haematological disease	0
**Onset symptoms**	
Sudden-onset, severe headache	0
Vomiting	0
Neck pain or stiffness	0
Photophobia	0
Blurred or double vision	0
Brief loss of consciousness	0
Seizures	0
**Clinical presentation on admission**	
GCS score	0
Focal neurological deficits	0
**Neuroimaging findings on admission**	
Location of blood within the subarachnoid space	
Basal cistern	4
Sylvian fissure	2
Interhemispheric fissure	3

Interpeduncular fossa	3
Suprasellar cistern	3
Ambient cistern	3
Quadrigeminal cistern	3
IVH	0
ICH	0
Subdural haemorrhage	0
Hydrocephalus	0
Evans' index	23
Hypodense lesions on computed tomography	2
**Admission laboratory investigations**	
Platelets (G/L)	7
PT-INR	17
**Initial severity of aneurysmal SAH**	
Modified WFNS score	0
WFNS score	0
H&H score	0
Fisher score	1
**Aneurysm repairs and other treatments**	
No aneurysm repair	0
Endovascular coiling	0
Surgical clipping	0
Surgical hematoma evacuation	0
EVD	1
IVF	0
Nimodipine	52
**Complications**	
Rebleeding	4
DCI	6
Acute hydrocephalus	0
Hyponatremia	0
Seizures	0
Chronic hydrocephalus	104
Ventriculitis	46
Pneumonia	0
Urinary tract infection	0
**Clinical time course**	
Ictus to hospital arrival (hour)	0
Length of hospitalization (days)	0
**Clinical outcomes**	
Hospital discharge	0
Transferred to another hospital	0
Discharged to die	0
Deaths:	
Died in hospital	0
Died within 30 days of ictus	0
Died within 90 days of ictus	0
Neurological function:	

mRS score at hospital discharge	0
mRS score at 30 days of ictus	0
mRS at 90 days of ictus	0

Abbreviations

COPD: chronic obstructive pulmonary disease; **DCI**: delayed cerebral ischemia; **EVD**: external ventricular drainage; **GCS**: Glasgow Coma Scale; **H&H**: Hunt and Hess; **ICH**: intracerebral haemorrhage; **IVF**: intraventricular fibrinolysis; **IVH**: intraventricular haemorrhage; **MI**: myocardial ischemia; **PT-INR**: prothrombin time with international normalized ratio; **SAH**: subarachnoid haemorrhage; **WFNS**: World Federation of Neurological Surgeons.
