# Supplementary material for: Validation of the accuracy of the modified World Federation of Neurosurgical Societies subarachnoid hemorrhage grading scale for predicting the outcomes of patients with aneurysmal subarachnoid hemorrhage
Source: PLoS One. 2023 Aug 22;18(8):e0289267. doi: 10.1371/journal.pone.0289267 (PMC10443875; doi:10.1371/journal.pone.0289267)
Supplement: Attachment — Submitted filename: Response to Reviewers.docx [file pone.0289267.s003.docx]

Professor Martin Kieninger

Academic Editor

PLOS ONE

*June 24, 2023*

Dear Prof. Martin Kieninger,

On behalf of all authors, I am resubmitting herewith our revised manuscript entitled *“Validation of the accuracy of the modified World Federation of Neurosurgical Societies Subarachnoid Hemorrhage Grading Scale for predicting the outcomes of patients with aneurysmal subarachnoid hemorrhage”* (PONE-D-22-32313R2).

We sincerely appreciate the kind comments and points raised by the Editors and by the Reviewers. We have carefully considered all comments and suggestions and revised our manuscript following each of these points. These comments have enabled us to substantially improve our manuscript. We hope that Editor will find our revised manuscript suitable for publication in PLOS ONE.

We confirm that this work is original and has not been published elsewhere nor is it currently under consideration for publication elsewhere. All authors have read, approved the manuscript, and agreed to authorship and order of authorship for this manuscript, and all authors have the appropriate permissions and rights to the reported data.

We have provided our point-by-point responses to the comments of the Editors and the Reviewers attached.

We thank you for your kind consideration of this submission.

Sincerely yours,

Chinh Quoc Luong, MD., PhD.

Center for Emergency Medicine,

Bach Mai Hospital,

No. 78, Giai Phong, Phuong Mai ward, Dong Da district, Hanoi 100000, Vietnam

Email: [luongquocchinh@gmail.com](mailto:luongquocchinh@gmail.com)

We thank the Editors and the Reviewers for the valuable comments and suggestions that greatly helped us to improve the contents of this paper. In what follows, we will use the **boldface** to indicate comments from the Editors and the Reviewers, the standard font face for our responses and we highlighted in yellow the modifications that we did to the manuscript.

**RESPONSE TO EDITORS**

**Thank you for submitting your manuscript to PLOS ONE. After careful consideration, we feel that it has merit but does not fully meet PLOS ONE’s publication criteria as it currently stands. Therefore, we invite you to submit a revised version of the manuscript that addresses the points raised during the review process.**

Our answer:

We thank you for the positive feedback. We have carefully considered the Reviewers' comments and suggestions and have revised our manuscript following each of these points.

**Please submit your revised manuscript by Jul 24 2023 11:59PM. If you will need more time than this to complete your revisions, please reply to this message or contact the journal office at plosone@plos.org. When you're ready to submit your revision, log on to https://www.editorialmanager.com/pone/ and select the 'Submissions Needing Revision' folder to locate your manuscript file.**

Our answer:

Thank you for this comment. We have submitted the revised manuscript on time.

**Please include the following items when submitting your revised manuscript:**

- **A rebuttal letter that responds to each point raised by the academic editor and reviewer(s). You should upload this letter as a separate file labeled 'Response to Reviewers'.**
- **A marked-up copy of your manuscript that highlights changes made to the original version. You should upload this as a separate file labeled 'Revised Manuscript with Track Changes'.**
- **An unmarked version of your revised paper without tracked changes. You should upload this as a separate file labeled 'Manuscript'.**

Our answer:

Thank you for this comment. We have included a rebuttal letter, a marked-up copy of the manuscript, and an unmarked version of the revised manuscript when submitting our revised manuscript.

**If you would like to make changes to your financial disclosure, please include your updated statement in your cover letter. Guidelines for resubmitting your figure files are available below the reviewer comments at the end of this letter.**

Our answer:

Thank you for this comment. We do not have any changes in our financial disclosure.

**If applicable, we recommend that you deposit your laboratory protocols in protocols.io to enhance the reproducibility of your results. Protocols.io assigns your protocol its own identifier (DOI) so that it can be cited independently in the future. For instructions see: https://journals.plos.org/plosone/s/submission-guidelines#loc-laboratory-protocols. Additionally, PLOS ONE offers an option for publishing peer-reviewed Lab Protocol articles, which describe protocols hosted on protocols.io. Read more information on sharing protocols at https://plos.org/protocols?utm_medium=editorial-email&utm_source=authorletters&utm_campaign=protocols.**

Our answer:

Thank you for this comment. Laboratory protocol does not apply to our study, but study protocol does. Our study protocol has included in the Methods section.

**We look forward to receiving your revised manuscript.**

**Kind regards,**

**Martin Kieninger**

**Academic Editor**

**PLOS ONE**

Our answer:

Thank the Editor and Reviewers so much for their support and for taking the time to leave their excellent reviews.

**Journal Requirements:**

**Please review your reference list to ensure that it is complete and correct. If you have cited papers that have been retracted, please include the rationale for doing so in the manuscript text, or remove these references and replace them with relevant current references. Any changes to the reference list should be mentioned in the rebuttal letter that accompanies your revised manuscript. If you need to cite a retracted article, indicate the article’s retracted status in the References list and also include a citation and full reference for the retraction notice.**

Our answer:

Thank you for this comment. We have reviewed the reference list, which is complete and correct. There are not any retracted articles on the list.

**RESPONSE TO REVIEWERS**

**Reviewers' comments:**

**Reviewer #3: The authors have in fact carefully addressed all aspects I have raised in my review. I congratulate the authors for this important study**

Our answer:

Thank the Reviewer so much for taking the time to leave his/her excellent review.

**Reviewer #4: Dear Dr. Luong. You and the manuscript writing team have made a great and highly precious work. I am really impressed by the study methodology as compared to the original WFNS, HH and MWFNS comparative reviews. The inclusion of all radiologic and epidemiological and clinical data, the transparent way of sample calculation and cut-off estimation as well as the logistic regression analysis were highly precise and well-performed. However, I would like the inclusion of 2 items to enhance the value of this great work:**

Our answer:

Thank the Reviewer so much for taking the time to leave his/her excellent review. We have carefully considered the Reviewer’s comments and suggestions and have revised our manuscript following each of these points.

**1- inclusion of PAASH scale in the calculation for ROC analysis curve and the cut-off prognostication table. the 5-category Prognosis on Admission of Aneurysmal Subarachnoid Hemorrhage (PAASH) grading scale has been shown by S.M. Dorhout Mees et al [DOI: 10.1161/STROKEAHA.107.498345] to show a more gradual increase of OR in ascending categories as compared to WFNS scale and as comparable to modified WFNS.**

Our answer:

Thank you for this valuable comment and suggestion. This study is part of an ongoing research project that aims to develop and validate the accuracy of several subarachnoid hemorrhage (SAH) grading scales based on admission Glasgow Coma Score (GCS) for predicting the outcomes of patients with aneurysmal SAH. With the latest updated dataset, we have performed further analysis and found that the 5-category Prognosis on Admission of Aneurysmal Subarachnoid Hemorrhage (PAASH) grading scale showed a more gradual increase of odds ratio in ascending categories compared to the Hunt and Hess (H&H), World Federation of Neurological Surgeons (WFNS), and modified WFNS grading scales. We will report the result in another paper.

**In your work, your stated that " the modified WFNS scale did not show more gradual increases in OR for the 30- and 90-day poor outcome, in ascending grades, compared to the WFNS and H&H scales ", so inclusion of PAASH gradual OR prognostication to this context will render your manuscript more comprehensive and recognizable. Additionally, the mRS 4-5 were defined by you as a poor outcome, though some papers include mRS 3 as well. An explanation for the situation of mRS 3 patient categories will be highly valuable. These notes don't demerit your valuable work. Rather, they ensure more worldwide recognition of it. Thank you.**

Our answer:

Thank you for your valuable comment and suggestion. This study is part of an ongoing research project that aims to develop and validate the accuracy of several subarachnoid hemorrhage (SAH) grading scales based on admission Glasgow Coma Score (GCS) for predicting the outcomes of patients with aneurysmal SAH. In this study, we defined good outcomes as a modified Rankin Scale (mRS) score of 0 to 3, which is also used in previously published studies.[1],[2] However, with the latest updated dataset, we will define excellent outcomes as an mRS score of 0 to 2 and report the result in another paper.

[1] Hoh BL, Ko NU, Amin-Hanjani S, Hsiang-Yi Chou S, Cruz-Flores S, Dangayach NS, Derdeyn CP, Du R, Hänggi D, Hetts SW, Ifejika NL, Johnson R, Keigher KM, Leslie-Mazwi TM, Lucke-Wold B, Rabinstein AA, Robicsek SA, Stapleton CJ, Suarez JI, Tjoumakaris SI, Welch BG. 2023 Guideline for the Management of Patients With Aneurysmal Subarachnoid Hemorrhage: A Guideline From the American Heart Association/American Stroke Association. Stroke. 2023 May 22. doi: 10.1161/STR.0000000000000436.

[2] Post R, Germans MR, Tjerkstra MA, Vergouwen MDI, Jellema K, Koot RW, Kruyt ND, Willems PWA, Wolfs JFC, de Beer FC, Kieft H, Nanda D, van der Pol B, Roks G, de Beer F, Halkes PHA, Reichman LJA, Brouwers PJAM, van den Berg-Vos RM, Kwa VIH, van der Ree TC, Bronner I, van de Vlekkert J, Bienfait HP, Boogaarts HD, Klijn CJM, van den Berg R, Coert BA, Horn J, Majoie CBLM, Rinkel GJE, Roos YBWEM, Vandertop WP, Verbaan D; ULTRA Investigators. Ultra-early tranexamic acid after subarachnoid haemorrhage (ULTRA): a randomised controlled trial. Lancet. 2021 Jan 9;397(10269):112-118. doi: 10.1016/S0140-6736(20)32518-6.

Thank the Editors and Reviewers so much for taking the time to leave their excellent reviews!

Sincerely yours,

Chinh Quoc Luong, MD., PhD.

Center for Emergency Medicine,

Bach Mai Hospital,

No. 78, Giai Phong, Phuong Mai ward, Dong Da district, Hanoi 100000, Vietnam

Email: [luongquocchinh@gmail.com](mailto:luongquocchinh@gmail.com)

On behalf of all authors
